# JNK and p53 cause human and mouse **β** cell death during excessive unfolded protein response

**DOI:** 10.1172/JCI193035

**Published:** 2026-08-03

**Authors:** Rohit B. Sharma, Christine Darko, Ying Wang, Thalia A. Castro, Tara Doma Lama, Brian Gablaski, Andrew Rappa, David Redmond, Jason K. Kim, Amy S. Lee, Laura C. Alonso

**Affiliations:** 1 Division of Endocrinology, Diabetes and Metabolism and the Joan and Sanford I. Weill Center for Metabolic Health, Weill Cornell Medicine, New York, New York, USA.; 2 Diabetes Division, UMASS Chan Medical School, Worcester, Massachusetts, USA.; 3 Ansary Stem Cell Institute, Division of Regenerative Medicine, Department of Medicine, Weill Cornell Medicine, New York, New York, USA.; 4 Program in Molecular Medicine, UMASS Chan Medical School, Worcester, Massachusetts, USA.; 5 Basic Science and Cancer Research, Keck School of Medicine, University of Southern California, Los Angeles, California, USA.

**Keywords:** Endocrinology, Metabolism, Cell stress, Diabetes, Insulin

## Abstract

Endoplasmic reticulum (ER) stress contributes to β cell death in both Type 1 and Type 2 diabetes (T1D and T2D). However, the molecular mechanisms driving β cell death during ER stress remain insufficiently defined, limiting development of protective therapies. GRP78, an ER chaperone, is the master regulator of unfolded protein response (UPR), suppressing UPR initiators during the unstressed state and releasing them to allow UPR activation during stress. To dissect the pathways leading to ER-stress response related β cell decompensation, we engineered mice genetically lacking GRP78 in pancreatic β cells. GRP78 deletion caused acute insulin-deficient diabetes in pups before weaning, with reduced β cell mass due to increased apoptosis. Molecular studies identified deregulated UPR, specifically IRE1 activity, as driving cell death. Unbiased and targeted analyses identified a JNK-p53 axis downstream of IRE1 kinase as a key mediator of β cell death during UPR activation. In vivo JNK inhibition protected against β cell death in 2 distinct ER stress diabetes models. In human β cells, pharmacological inhibition of both JNK and p53 improved β cell survival during GRP78 knockdown–induced UPR. These findings provide insight into mechanisms causing β cell death during ER stress and outline possible therapeutic targets to preserve insulin secretory capacity in diabetes.

## Abstract 2

This study identifies JNK and p53 as mediators of pancreatic beta cell death during ER stress, which could lead beta cell preserving therapies for diabetes.

## Graphical abstract

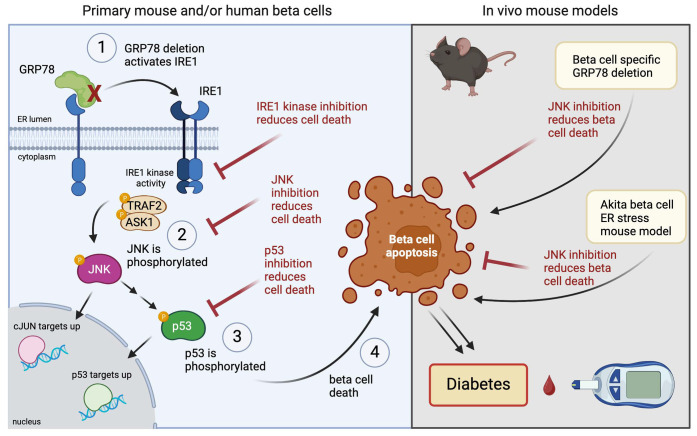

## Introduction

Pancreatic β cells are the sole source of circulating insulin. β cell dysfunction or loss leads to diabetes, whether due to autoimmune attack (type 1 diabetes, T1D) or in the context of obesity and insulin resistance (type 2 diabetes, T2D) (1). Insulin is a secreted polypeptide, and proinsulin synthesis places substantial demand on the β cell endoplasmic reticulum (ER) (2, 3). Active β cells dynamically engage the unfolded protein response (UPR), an ER stress response system, to adapt to insulin synthesis demand. As such, the UPR is a critical pillar for maintaining healthy β cell function (3). Paradoxically, however, not just unresolved ER stress but also excess UPR stress response can lead to β cell dysfunction and demise (4, 5).

The specific mechanisms responsible for β cell death during ER stress and the stress response are important, as they represent opportunities to intervene to prevent or treat both T1D and T2D by enhancing β cell survival (5, 6). The best understood mechanisms linking canonical ER stress pathways with cell death include excessive PERK activation, which leads to accumulation of prodeath C/EBP homologous protein (CHOP) (7) and IRE1 hyperactivation, which causes a nonspecific toxic degradation of ER-associated mRNAs called regulated IRE1-dependent decay (RIDD) (8). Other pathways leading to cell death have also been reported (4, 5). In addition, loss-of-function mutations in genes encoding ER proteins can cause β cell failure (9–17).

The 78-kDa glucose regulated protein (GRP78; BIP; encoded at *HSPA5*) is an abundant calcium-binding ER resident Hsp70-family chaperone discovered in 1977 as a protein induced by glucose starvation (18, 19). GRP78 has multiple functions, including chaperone activity, targeting misfolded proteins for ERAD, maintaining ER calcium stores, and, crucially, regulating the initiation of UPR pathways (20). Cellular GRP78 reduction leads to unregulated activation of the ER stress response. Deletion of GRP78 from all tissues in mice results in early embryo lethality around the time of implantation (21).

To explore mechanisms of β cell failure during ER stress with unresolved UPR, we engineered β cell–specific GRP78 deletion in mice. The results show GRP78 to be essential beyond its known role in proinsulin folding, as a critical factor without which β cells are lost en masse through apoptosis. Inhibiting UPR pathways rescued cell death, suggesting that apoptosis was due to unregulated UPR activation rather than unmet stress load. Tracing the molecular pathway causing β cell death revealed that, surprisingly, CHOP was not required; instead, a IRE1-JNK-p53 mechanism was responsible for β cell death. We identify acute inhibition of JNK and p53 as potential therapeutic approaches to preserve β cell mass during ER stress. This work provides insight into the molecular events leading to diabetes when β cell stress-resolving mechanisms fail.

## Results

### β cell–specific deletion of Grp78 causes early postnatal diabetes due to insulin deficiency.

**Figure 1:**
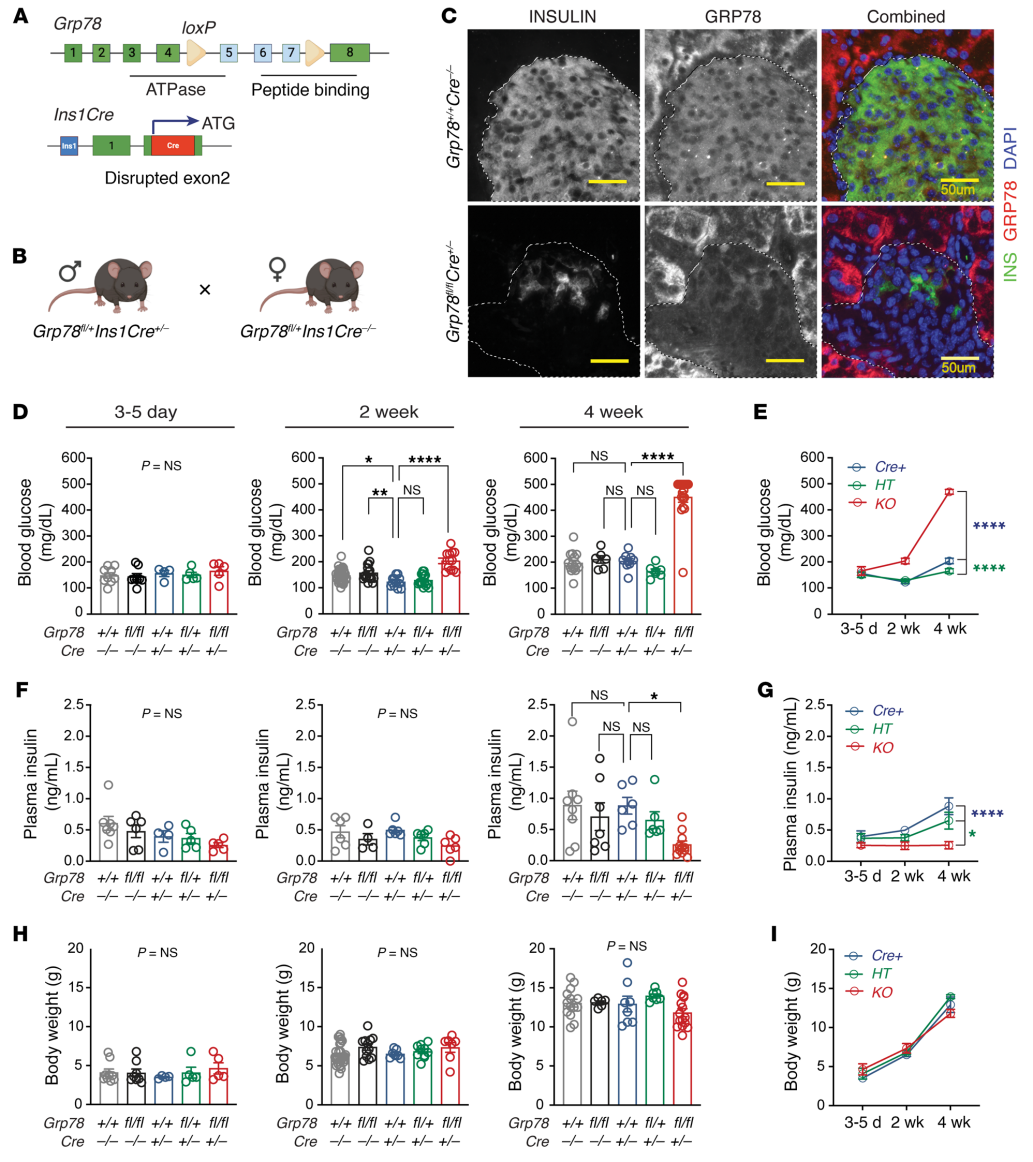
Deletion of key ER chaperone GRP78 from β cells in mice caused insulin-deficient diabetes within 2 weeks of life. (**A**) *Grp78* gene locus (not to scale) indicating the location of loxP sites and *Ins1cre* knock-in allele. Blue exons are removed after recombination. (**B**) Breeding strategy to generate β cell–specific *Grp78^fl/fl^Ins1cre^+/–^* mice and littermate controls. (**C**) Pancreas sections of 4-week old *Grp78^+/+^Ins1cre^+/–^* (Cre control) and *Grp78^fl/fl^Ins1cre^+/–^* (GRP78 deletion) immunostained for insulin (green), GRP78 (red) and DAPI (blue). (**D**–**I**) (*n* ≥ 4): nonfasting blood glucose (**D** and **E**), plasma insulin (**F** and **G**), and body weight (**H** and **I**) of all littermate genotypes. Males and females did not show differences for these outcomes and were combined. Scale bars: 50μm. Statistics by 1-way ANOVA. *P < 0.05; **P < 0.01; ***P < 0.001; ****P < 0.0001.

We generated β cell–specific GRP78 deletion by crossing *Grp78^fl/fl^* mice (21) with *Ins1cre* mice in which *Cre* is knocked in at the *Ins1* gene locus (*Ins1cre^+/–^*) (22). *Ins1cre* mice in our colony exhibit close to 100% recombination efficiency (Supplemental Figure 1; supplemental material available online with this article; https://doi.org/10.1172/JCI193035DS1). To avoid exposing pups to maternal β cell GRP78 or INS1 reduction in utero, *Grp78^fl/+^Ins1cre^+/–^* males were crossed to *Grp78^fl/+^Ins1cre^–/–^* females, which have 2 healthy *Ins1* alleles (Figure 1, A and B). GRP78 protein reduction in β cells was confirmed by immunostaining pancreas sections (Figure 1C).

We studied the metabolic health of progeny at 3–5 days, 2 weeks, and 4 weeks of age. Mice with β cell *Grp78* deletion had normal random blood glucose at 3–5 days of age, but glucose started to increase at 2 weeks and was markedly elevated at 4 weeks compared with littermate controls (Figure 1, D and E). Male and female *Grp78^fl/fl^Ins1cre^+/–^* mice were equally prone to diabetes (Supplemental Figure 2); therefore, combined data are shown. *Grp78* heterozygous mice were indistinguishable from controls. Since the intervention targeted β cells, we hypothesized that the hyperglycemia might be due to reduced insulin levels. Indeed, plasma insulin was significantly lower at 4 weeks in *Grp78*-deleted mice (Figure 1, F and G). Despite the severe hyperglycemia, body weight was not reduced in 4-week *Grp78*-deleted mice (Figure 1, H and I). To determine whether the diabetic phenotype was sustained into adulthood, *Grp78^fl/fl^Ins1cre^+/–^* mice and littermate controls were studied at 8 weeks of age (Supplemental Figure 3). *Grp78*-deleted mice showed evidence of ongoing catabolic state, with markedly reduced body weight and elevated blood glucose.

### Mice lacking β cell Grp78 have reduced β cell mass and disrupted islet architecture.

**Figure 2:**
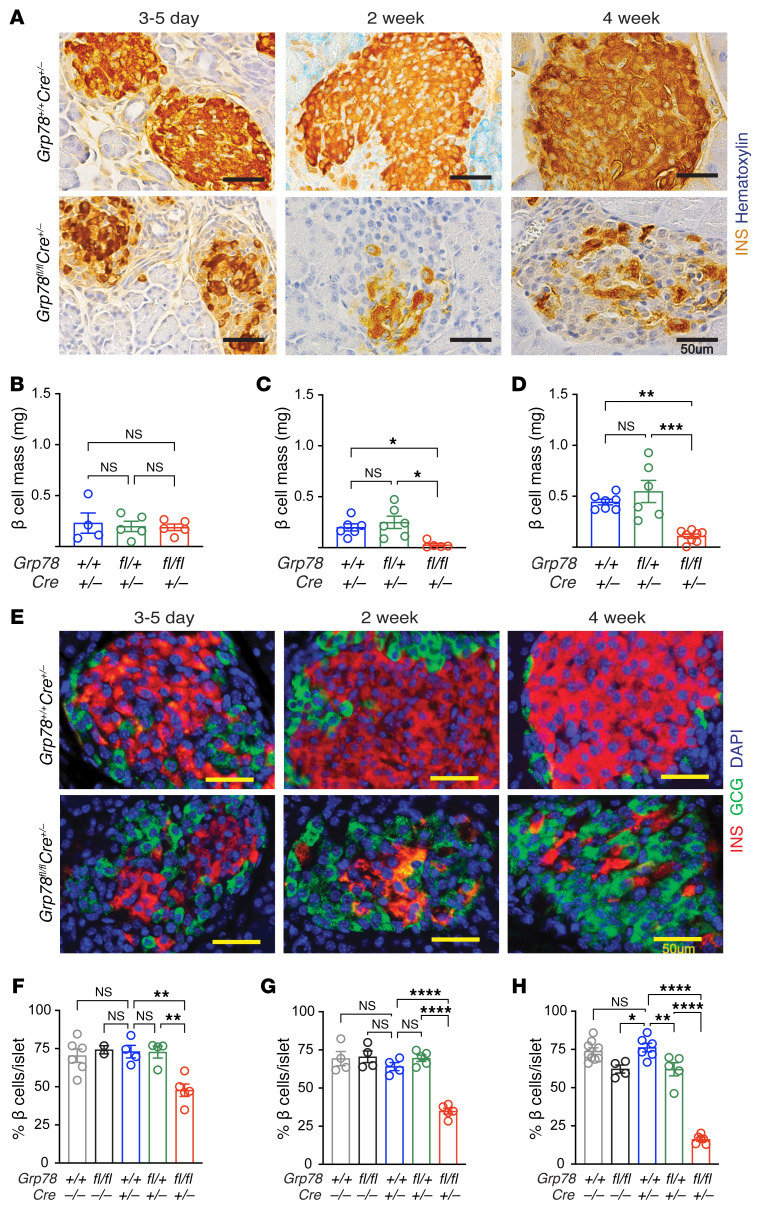
β cell GRP78 deletion reduced β cell mass and disrupted islet architecture. (**A**) IHC of pancreas sections for insulin (brown) and hematoxylin (blue) was used to estimate β cell mass (**B**–**D**) (*n* ≥ 4) of *Grp78^+/+^Ins1cre^+/–^* (cre control), *Grp7^f/+^Ins1cre^+/–^* (heterozygous) and *Grp78^fl/fl^Ins1cre^+/–^* (deletion) mice. (**E**) Immunostaining of pancreas sections for insulin (red), glucagon (green), and DAPI (blue) was used to quantify the percentage islet endocrine cells that labeled for insulin (**F**–**H**) (*n* ≥ 2). Males and females were combined. Scale bars: 50μm. Statistics by 1-way ANOVA. *P < 0.05; **P < 0.01; ***P < 0.001; ****P < 0.0001.

Hyperglycemia due to insulin deficiency in β cell specific *Grp78*-deleted mice could be due to reduction in β cell mass, insulin secretory function, or both. To measure β cell mass, pancreas sections were immunostained for insulin and counterstained with hematoxylin (Figure 2A). Islets from *Grp78*-deleted mice had visibly fewer insulin-labeled cells than littermate controls at 2 and 4 weeks, with loss of nearly all β cells by 8 weeks of age (Supplemental Figure 3). Pancreas weight was similar across genotypes at 4 weeks (data not shown), but reduced at 8 weeks of age, proportionate to the reduction in body size. Quantification identified a pronounced reduction in the percentage of insulin-positive area (data not shown) and β cell mass (Figure 2, B–D) in *Grp78*-deleted mice compared with control genotypes by 2 weeks of age (Figure 2C), which deepened at 4 weeks of age (Figure 2D) when control mice started to meaningfully expand β cell mass.

Islets in *Grp78*-deleted mice contained many insulin-negative cells, suggesting an altered cellular composition that might be due to a relative increase in other endocrine cells. Immunostaining for insulin and glucagon revealed a striking disruption of islet architecture. While control mice had a typical murine islet structure with peripheral α cells surrounding a β cell core, *Grp78*-deleted islets had relatively more α cells, fewer β cells, and an abnormal distribution of α cells in the islet center (Figure 2E). Quantification of insulin- and glucagon-labeled cells (Figure 2, F–H) showed a significant reduction in percentage β cells per islet as early as 3–5 days of age in *Grp78*-deleted pups (Figure 2F), before the reduction in β cell mass was evident, which worsened over time such that islets of 4-week *Grp78*-deleted mice contained only 20% β cells (Figure 2, G and H). Assessments of non–β islet cells (Supplemental Figures 4 and 5) revealed that α cell proliferation was not increased, nor was absolute α cell mass, suggesting the increased proportion of α cells might be passive, due to loss of β cells, rather than active expansion of the α cell compartment. Somatostatin-labeled cells were also increased. Intriguingly, the proportion of islet cell labeling for both insulin and glucagon was increased, which could represent stress-related α-to-β or β-to-α transdifferentiation (23), or failure of full maturation in this early-life time frame. Since there was no meaningful recovery of β cell mass at 8 weeks, any cellular plasticity this might represent did not rescue β cell number. Circulating glucagon levels were not elevated (Supplemental Figure 4F).

### Loss of β cell mass is primarily due to increased β cell apoptosis.

**Figure 3:**
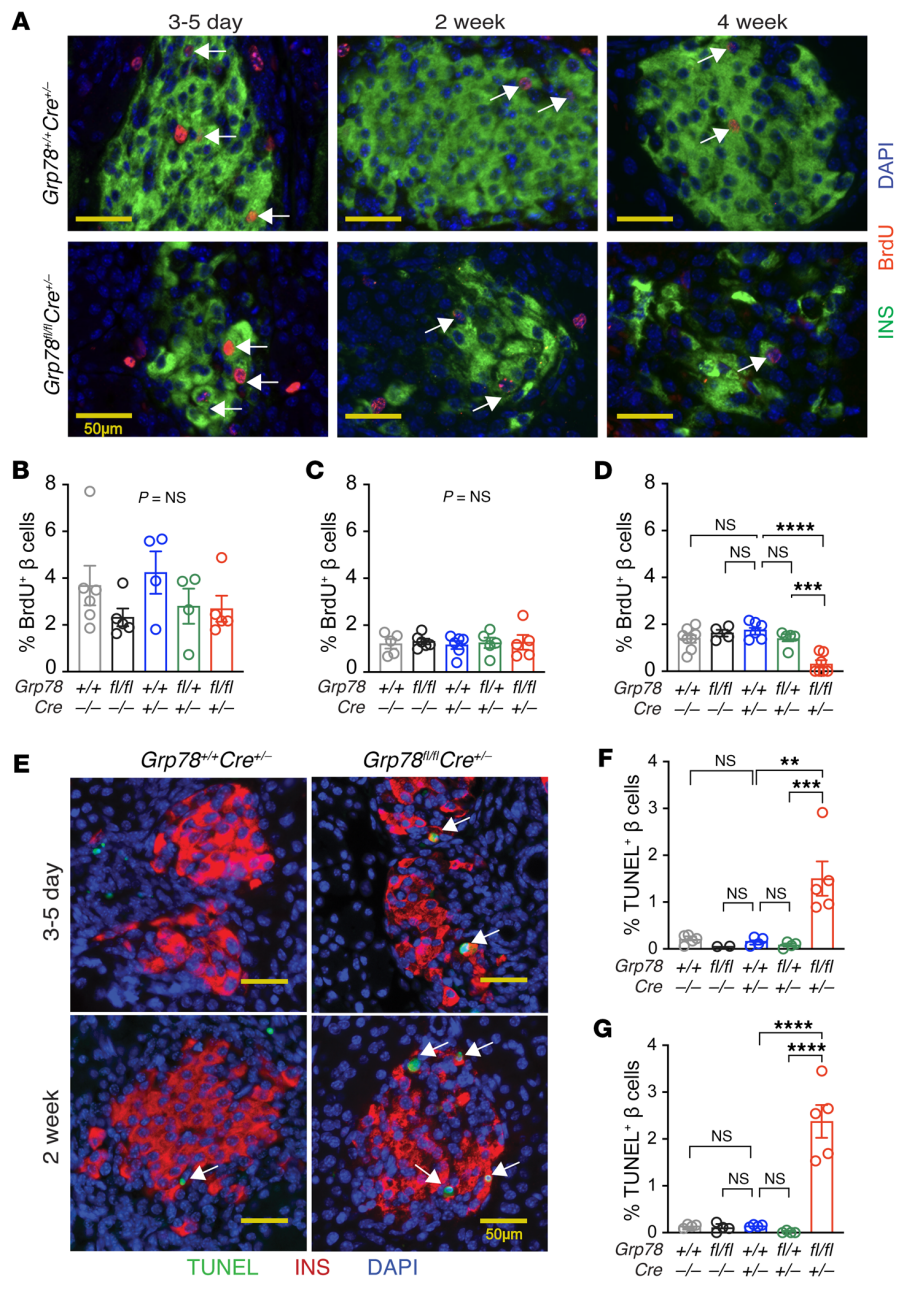
Loss of β cell mass after GRP78 deletion was predominantly due to increased β cell death. (**A**) Immunostaining of pancreas sections from 3–5 day, 2-week and 4-week old mice for insulin (green), BrdU (red), and DAPI (blue). (**B**–**D**) Quantitation of the percentage of insulin-positive cells that were also BrdU-positive from all genotypes at 3–5 days (left), 2 weeks (center), and 4 weeks (right) (*n* ≥ 4). (**E**) TUNEL (green) labeling of pancreas sections from 3–5 day and 2-week old mice as well as for insulin (red), and DAPI (blue). (**F** and **G**) Quantitation of the percentage of insulin^+^ cells that were also TUNEL-positive in 3–5 day (**F**) and 2-week (**G**) mice (*n* ≥ 2). White arrows in **A** and **E** point to examples of dual-positive cells. Males and females combined. Scale bars: 50μm. Statistics by 1-way ANOVA. *P < 0.05; **P < 0.01; ***P < 0.001; ****P < 0.0001.

The collapse of the β cell population during early postnatal development could be due to loss of proliferative expansion, premature cell death, or both. Quantification of pancreas sections immunostained for insulin and S-phase label bromodeoxyuridine (BrdU; administered in 2 injections 4 and 2 hours before euthanasia) (Figure 3A) showed that β cell proliferation was indeed reduced in *Grp78*-deleted mice, but not until 4 weeks of age, well after the β cell loss began (Figure 3, B–D). This suggested that reduced proliferation might contribute to the accelerated loss of β cell mass that occurs after 2 weeks of age but was not likely the cause of the β cell deficit observed between birth and 2 weeks. In contrast, labeling β cells for apoptosis showed an evident excess of TUNEL-positive β cells in *Grp78-*deleted pups (Figure 3E) as early as 3–5 days of age (Figure 3, F and G). Taken together, these data suggest that reduced β cell mass accrual after birth in mice lacking *Grp78* in β cells is due predominantly to an early increase in β cell death, with late reduction in proliferation as a secondary contributor.

### GRP78 deletion induces a transcriptional signature of pathways involved in β cell death.

**Figure 4:**
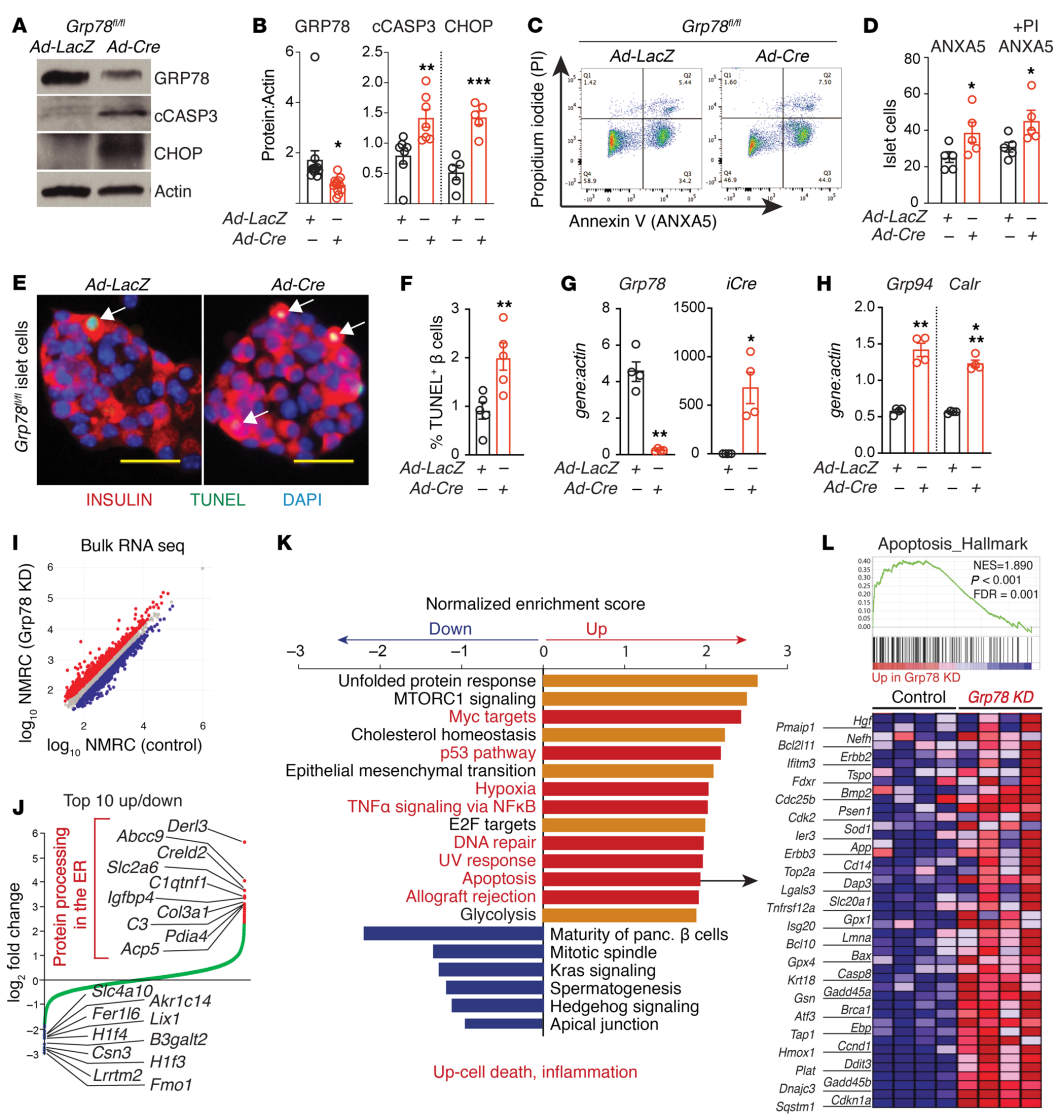
Ex vivo GRP78 depletion increased β cell death and transcriptional signatures of apoptosis pathways. Dispersed *Grp78^fl/fl^* male and female mouse islet cells were transduced with *Ad-LacZ* (control) or *Ad-Cre* (knockdown, KD) and cultured for 72 hours in 15 mM glucose for all experiments in this figure. (**A** and **B**) Western blots for GRP78, cleaved caspase 3 (cCASP3), CHOP, and Actin (**A**) were quantitated in **B** (*n* ≥ 5). (**C** and **D**) Cells were lifted, stained for Annexin V (ANXA5) and propidium iodide (PI), analyzed by flow cytometry (**C**), and quantitated (**D**) (*n* ≥ 5). (**E** and **F**) Cells plated on glass coverslips were stained for insulin (red), TUNEL (green) and DAPI (blue), imaged (**E**), and dying β cells were counted (**F**) (*n* ≥ 4). (**G** and **H**) qPCR analysis on parallel aliquots of RNA to those sent for library preparation and sequencing were tested for *Grp78* (**G**) or compensatory chaperones *Grp94* and *Calr* (**H**) (*n* = 4). (**I**–**L**) Bulk RNA sequencing of islet cell cultures prepared as above; *n* = 4 biological replicates (2 male, 2 female). (**I**) Scatter plot of differentially expressed genes (*P* < 0.05, FC ≥ 1.5); red dots are upregulated in *Grp78* KD, blue dots are downregulated in *Grp78* KD. (**J**) Log_2_ fold change with top 10 up- and downregulated genes labeled. (**K**) Illustration of significantly enriched (FDR < 0.05) upregulated (red, related to cell death) and downregulated (blue) Hallmark gene sets. (**L**) Enrichment plot and heat map of hallmark apoptosis gene set showing gene expression changes. Scale bars: 50 μm. Statistics by paired *t* test. *P < 0.05; **P < 0.01; ***P < 0.001; ****P < 0.0001.

To dissect the mechanism of β cell death caused by reduction in GRP78, we developed an ex vivo model of *Grp78* depletion by isolating pancreatic islets from healthy *Grp78^fl/fl^* mice and introducing *Cre* by adenovirus to acutely knock down *Grp78* ex vivo in dispersed islet cells. Quantitative PCR analysis indicated robust *iCre* expression, marked *Grp78* mRNA reduction, and compensatory induction of *Grp94* chaperone, which indicates functional loss of GRP78 protein (Supplemental Figure 6). An immunoblot performed 72 hours after transduction showed a partial reduction in GRP78 protein levels (Figure 4, A and B). As with in vivo deletion, ex vivo *Grp78* knockdown increased β cell death, as measured by induction of terminal cell death executor protein cleaved caspase 3 (cCASP3) and ER stress cell death driver CHOP (Figure 4, A and B); by flow cytometry labeling for annexin V and PI (Figure 4, C and D); and by TUNEL staining in insulin-positive cells (Figure 4, E and F).

To explore mechanisms leading to β cell death after GRP78 depletion, we performed bulk RNA-seq on dispersed primary islet cells from 4 adult *Grp78^fl/fl^* mice (2 females, 2 males) after 72 hours of ex vivo knockdown by transducing with *Ad-LacZ* (control) or *Ad-Cre* (knockdown), a time point when β cell death had begun, but the cultures still contained mostly β cells. qPCR of parallel aliquots of the samples submitted for sequencing confirmed *Grp78* knockdown and compensatory increase in *Grp94* and *Calr* (Figure 4, G and H). RNA-seq revealed altered expression of 1,672 genes (835 up, 837 down; *P* < 0.05, FC > 1.5; Figure 4, I and J). Encouragingly, Hallmark gene set enrichment analysis identified UPR activation as the topmost enriched pathway (Figure 4K). Of the upregulated gene sets with FDR < 0.05, 8 sets were related to cell death, cell damage, and inflammation, confirming the importance of GRP78 in cellular integrity. Some upregulated gene sets (Myc targets, E2F targets, glycolysis) suggested the possibility that Grp78 knockdown might increase β cell proliferation; however, ex vivo studies did not confirm this hypothesis (Supplemental Figure 7). Many upregulated genes were associated with apoptotic cell death (Figure 4L). The top downregulated gene set was “maturity of pancreatic β cells,” suggesting the possibility that GRP78 depletion may lead to loss of β cell function, with the caveat that the intervention caused β cell death, and bulk RNA-seq is susceptible to alterations in cell type abundance in the sequenced material. Overall, the RNA-seq experiment provided strong evidence that GRP78 depletion activates specific molecular pathways leading to β cell death and uncovered clues as to the nature of those pathways.

### Depletion of GRP78 causes β cell death via IRE1 and JNK phosphorylation.

A key role of GRP78 under unstressed conditions is to suppress the activation of UPR initiators PERK, IRE1, and ATF6, and the RNA-seq revealed UPR as the highest upregulated gene set after GRP78 depletion in islet cell cultures. Indeed, some genes in the apoptosis gene set induced by GRP78 knockdown were identified as UPR-linked, including *Ddit3* and *Atf3,* both downstream of PERK activation.

**Figure 5:**
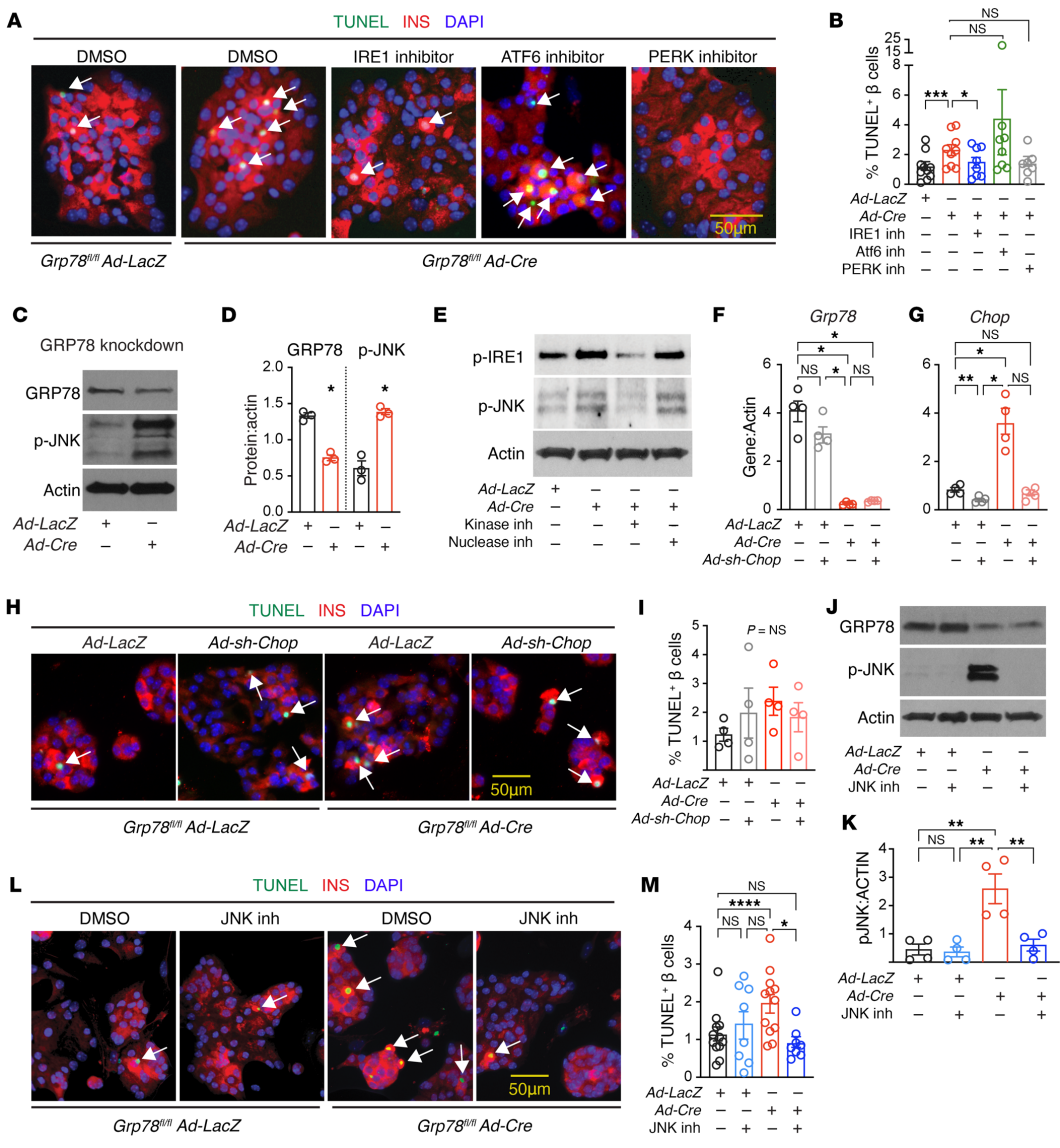
IRE1-dependent JNK activation, but not CHOP induction, leads to β cell death after GRP78 depletion. Dispersed *Grp78^fl/fl^* male and female mouse islet cells were transduced with *Ad-LacZ* or *Ad-Cre* and cultured for 72 hours in 15 mM glucose for all panels. (**A** and **B**) Cultures were labeled for TUNEL (green), insulin (red), and DAPI (blue) after treatment with inhibitors of IRE1 (4μ8c), ATF6 (AEBSF), or PERK (GSK2606414) with or without GRP78 knockdown (*n* ≥ 7). Inhibitors were added for the full 72 hours. See Supplemental Figure 3 for *Ad-LacZ* data with inhibitors. (**C** and **D**) Immunoblot of GRP78, p-JNK, and actin 72 hours after GRP78 knockdown (*n* ≥ 4). (**E**) Immunoblot of p-IRE1, p-JNK, and actin after GRP78 knockdown in the absence or presence of IRE1 inhibitors specific for the kinase or nuclease activity. (**F** and **G**) qPCR for *Grp78* or *Chop* on GRP78 knockdown islet cultures with or without transduction with *Ad-sh-Chop* (*n* = 4). (**H** and **I**) GRP78 knockdown islet cell cultures transduced with *Ad-sh-Chop* and labeled for TUNEL (green), insulin (red) and DAPI (*n* = 4). For **F**–**I**, total virus MOI was held constant for all conditions by adjusting the MOI of *Ad-LacZ*. (**J** and **K**) Immunoblot and band intensity quantitation of GRP78, p-JNK, and actin 72 hours after GRP78 knockdown in the presence or absence of JNK inhibitor (JNK-IN-8) (*n* = 4). (**L** and **M**) GRP78 knockdown islet cell cultures treated with pan-JNK inhibitor labeled for TUNEL (green), insulin (red) and DAPI (*n* ≥ 8). Scale bars: 50μm. Statistics by paired *t* test (**D**) or 1-way ANOVA (**B**, **F**, **G**, **I**, **K**, and **M**). *P < 0.05; **P < 0.01; ***P < 0.001; ****P < 0.0001.

We hypothesized that the β cell death observed after GRP78 knockdown might be related to unrestrained UPR activation. Confirming the RNA-seq profile, all 3 canonical UPR pathways were activated at the mRNA level after *Grp78* knockdown in islet cells (Supplemental Figure 6). Since UPR pathway activation can paradoxically increase cell death by multiple mechanisms (4), we measured β cell death after GRP78 depletion while inhibiting each of the 3 UPR initiators. TUNEL staining was performed on mouse islet cells treated with inhibitors of ATF6 (AEBSF), IRE1 (4μ8c), or PERK (GSK2606414) applied for the 72-hour duration of *Grp78* knockdown (Figure 5A). Under control conditions without *Grp78* knockdown, PERK and IRE1 inhibition did not impact β cell death, but ATF6 inhibition tended to increase β cell death (Supplemental Figure 8). The impression of increased cell death was reinforced by a marked reduction in cell number, leaving few cells to count. This result suggested that acute ATF6 inhibition precipitates cell death in dispersed mouse islet cell cultures even if GRP78 remains intact.

Importantly, inhibition of IRE1 reversed the increased β cell death after GRP78 depletion (Figure 5, A and B). PERK inhibition also reduced cell death, although the result was not statistically significant. Taken together, these data suggest that ATF6 plays a prosurvival role in mouse islet cell cultures and that IRE1 and possibly PERK contribute to β cell death when GRP78 is depleted.

Two prominent mechanisms by which ER stress leads to cell death are by activating IRE1 kinase activity to phosphorylate TRAF2, which activates JNK (24) via phosphorylation, or by activating PERK to phosphorylate eIF2α, enhancing ATF4 translation, which increases the abundance of CHOP (7). GRP78 depletion in islet cell cultures strongly increased phosphorylation of JNK (Figure 5, C and D) and increased the abundance of CHOP (Figure 4, A and B), suggesting that both mechanisms might be active. JNK phosphorylation was lost in the presence of an IRE1 kinase inhibitor (APY29) but remained intact in the presence of an IRE1 nuclease inhibitor (STF-083010), confirming that JNK activation after GRP78 depletion was dependent on IRE1 kinase activity (Figure 5E).

To explore the roles of CHOP and JNK in β cell death after GRP78 depletion, we tested *Chop* knockdown via shRNA, or JNK inhibition via a well-established small molecule inhibitor JNK-IN-8 (pan JNK inhibitor) (25). *Ad-sh-Chop* effectively reduced *Chop* expression (Figure 5, F and G), but surprisingly did not protect against β cell death after GRP78 depletion (Figure 5, H and I). On the other hand, the JNK-In-8 inhibitor, which reduced JNK phosphorylation induced by GRP78 knockdown (Figure 5, J and K), fully rescued cell death after GRP78 depletion (Figure 5, L and M). Taken together, these data suggest that IRE1-dependent activation of p-JNK, but not CHOP, is responsible for increased β cell death following loss of GRP78.

### JNK-dependent p53 activation leads to β cell death after Grp78 knockdown.

**Figure 6:**
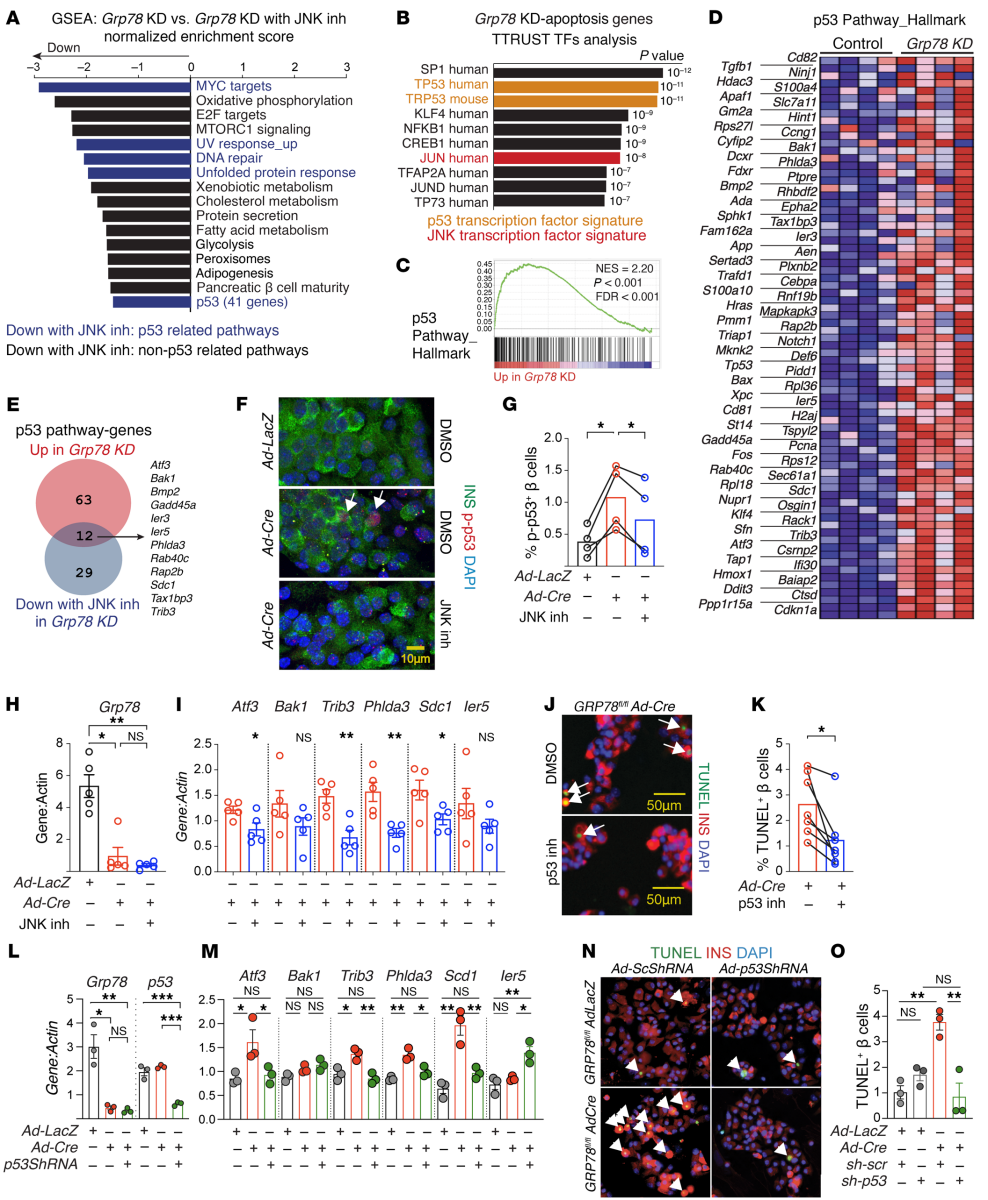
JNK inhibition or acute p53 inhibition both rescue β cell death. All panels show experiments on dispersed *Grp78^fl/fl^* mouse islet cells cultured for 72 hours in 15 mM glucose. (**A**) GSEA for hallmark gene sets on RNA-seq of *Grp78* knockdown (KD) mouse islet cells with or without JNK inhibition. Only downregulated gene sets are show. Blue, p53-related gene sets. (**B**) TTRUST transcription factor prediction analysis on apoptosis gene set genes upregulated by *Grp78* knockdown. Orange, p53-related; red, JNK-related. (**C**) Enrichment plot, p53 hallmark genes upregulated after Grp78 knockdown. (**D**) Heatmap, p53 hallmark genes upregulated after Grp78 knockdown. (**E**) Venn diagram showing the overlap of p53 genes upregulated by reducing GRP78 and downregulated by JNK inhibition during GRP78 knockdown. (**F** and **G**) Islet cells immunostained for insulin (green), phospho-p53 (red), and DAPI (blue) imaged by confocal microscopy, percentage β cells with nuclear phospho-p53 (*n* = 4). (**H** and **I**) qPCR analysis for *Grp78* (**H**) or p53 transcriptional targets *Atf3, Bak1, Trib3, Phlda3, Sdc1,* and *Ier5* (*n* = 5). (**I**). (**J** and **K**) Islet cells with Grp78 knockdown with or without p53 inhibition were fixed, labeled for insulin (red), TUNEL (green), and DAPI (blue), imaged, and counted (*n* = 7). (**L**–**O**) Islet cells withGrp78 knockdown with or without p53 knockdown underwent qPCR for Grp78, p53, or p53 target genes (**L** and **M**), or TUNEL staining and quantification (**N** and **O**), *n* = 3. Scale bars: 50μm. Statistics by paired *t* test (**H** and **J**) or 1-way ANOVA (**F** and **G**). *P < 0.05; **P < 0.01.

To explore how JNK activation leads to β cell death, we performed a second RNA-seq experiment, now testing for gene expression changes in mouse islet cells after JNK inhibition in the setting of *Grp78* knockdown. GRP78-depleted islet cells exposed to JNK inhibitor, compared with vehicle control, resulted in only 194 gene changes (141 up, 53 down, *P* < 0.05, FC = 1.2, data not shown). We hypothesized that JNK-dependent pathways leading to cell death after GRP78 depletion might be identified as those death-related gene sets that were upregulated by *Grp78* knockdown but downregulated by JNK inhibition during *Grp78* knockdown. Comparing the gene sets downregulated after JNK inhibition (Figure 6A) with those upregulated after loss of GRP78 (Figure 4K), we found several common pathways, including Myc targets, UV response, UPR, and p53.

Separately, we sought to identify transcriptional regulators of the apoptosis genes activated by *Grp78* knockdown. Intriguingly, exploring the GRP78-depletion apoptosis gene signature (Figure 4L) using the TRRUST transcription factor prediction tool independently suggested p53 as a driver of the apoptosis phenotype induced by GRP78 deletion (Figure 6B). Indeed, going back to the initial RNA-seq experiment, many p53 pathway genes were upregulated by GRP78 knockdown (Figure 6, C and D), and 29% (12 of 41) of the p53 pathway genes reduced by JNK inhibition overlapped with genes upregulated with the knockdown of GRP78 (Figure 6E). JNK is a stress kinase with many substrates, including p53 (26), and phosphorylation of p53 stabilizes it to maintain it in the nucleus (27). p53 is a well-known tumor suppressor that increases cell death in many cell types (28), including β cells (29–31). Taken together, these observations suggested that GRP78 depletion might increase cell death through JNK activation of p53 and, conversely, that inhibiting JNK might reduce β cell death by inhibiting p53.

We thus hypothesized that during GRP78 depletion JNK might phosphorylate p53 in β cells, retaining phospho-p53 in the nucleus to activate its transcriptional targets and induce β cell death. To test this hypothesis, we first asked whether nuclear phospho-p53 was increased in β cells after GRP78 deletion, and if so, whether inhibiting JNK reversed this trend. Indeed, confocal microscopy showed that phospho-p53 was evident in some β cell nuclei in GRP78 knockdown conditions, and the effect was reduced by JNK inhibition (Figure 6, F and G). Furthermore, JNK inhibition reduced the abundance of p53 transcriptional targets (Figure 6, H and I), suggesting that p53 was less active if JNK was inhibited. However, canonical p53 target p21 (*Cdkn1a*), although induced by Grp78 knockdown (Figure 6D), was not impacted by JNK inhibition (data not shown).

Finally, we tested whether inhibiting p53 protected against β cell death caused by *Grp78* knockdown. Indeed, β cell death was reduced in islet cell cultures pretreated with the p53 inhibitor pifithrin α before GRP78 depletion (Figure 6, J and K). To assess whether acute reduction of p53 expression improved β cell survival, we performed genetic knockdown experiments using shRNA (Figure 6, L–O, and Supplemental Figure 9) in islet cell cultures with GRP78 depletion. p53 knockdown successfully reduced *p53* mRNA and prevented the induction of p53 transcriptional targets caused by *Grp78* knockdown (Figure 6, L and M). TUNEL experiments showed that p53 reduction prevented the increase in β cell death in these cultures (Figure 6, N and O), and qPCR analyses noted a reversal in *Bax*, *Bcl2*, and *Bcl-xl* without changing JNK gene target expression (Supplemental Figure 9). Taken together, these observations suggest that when GRP78 is depleted from healthy mouse β cells, JNK is activated, which leads to increased p53 transcriptional activity that contributes to β cell death. Importantly, acute p53 inhibition under these conditions improved β cell survival.

### In vivo JNK inhibition reduces β cell death in mice with GRP78 deletion.

**Figure 7:**
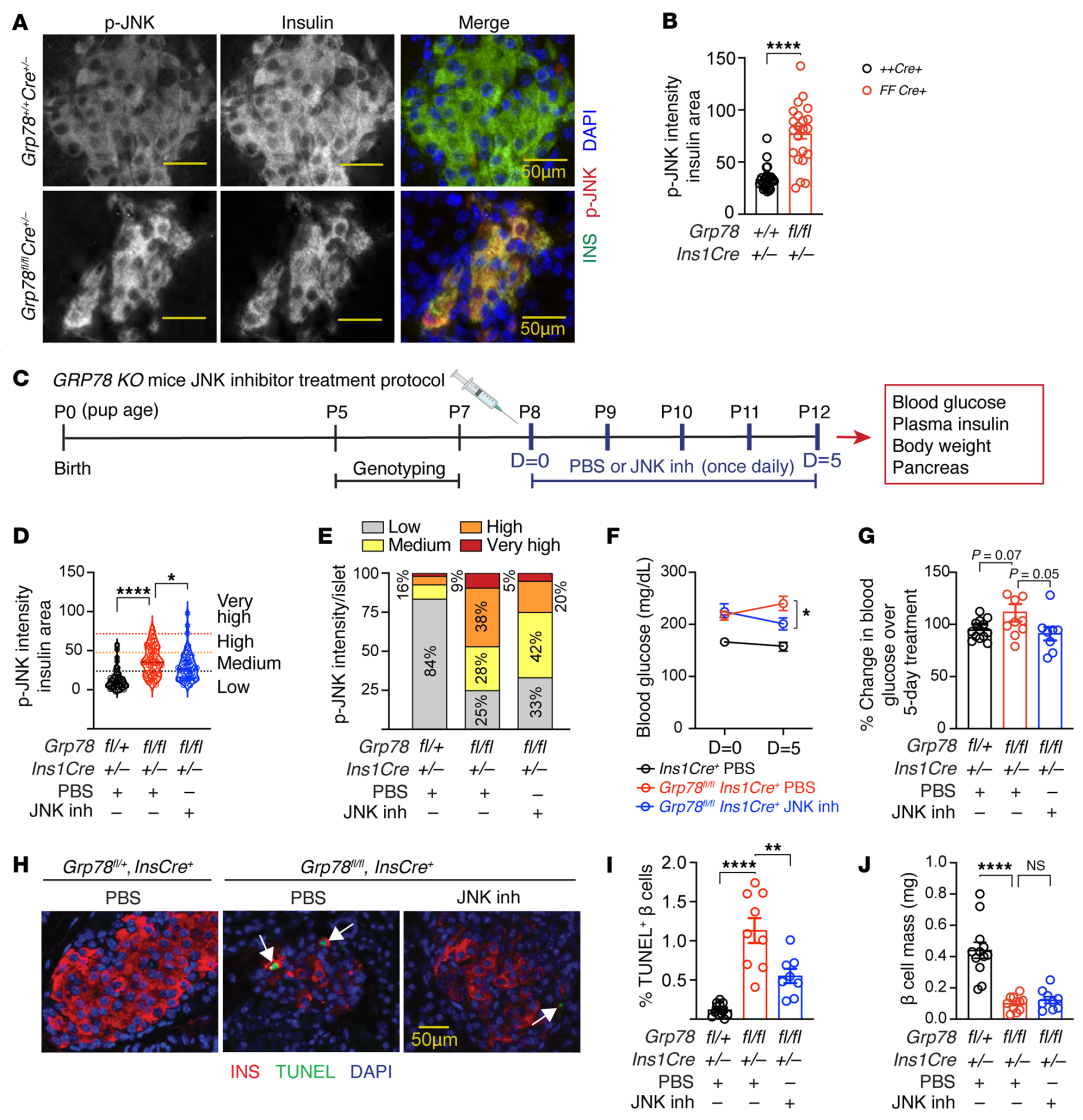
JNK inhibition reduces β cell death in *Grp78*-deletion mice. All panels show analyses of *Grp78^fl/fl^Ins1cre^+/–^* mice and littermate *Grp78^+/+^Ins1cre^+/–^* controls. (**A** and **B**) Pancreas sections of 2-week old mice immunostained for insulin (green), phospho-JNK (p-JNK, red), and DAPI (blue) were imaged (**A**) and quantified (**B**) for p-JNK intensity in insulin^+^ area using ImageJ. Each data point represents staining intensity across one islet; 3–10 islets were quantified from *n* = 3 mice of each genotype. (**C**) Schematic of in vivo treatment timeline and sample collection. JNK inhibitory peptide was injected intraperitoneally for 5 days from P8–P12. (**D** and **E**) Quantitation of pJNK intensity in *Grp78*-deletion mice or controls treated with PBS or JNK inhibitor; 10 islets from *n* = 5–6 mice of each treatment group were analyzed. Dotted lines in **D** denote demarcations between expression intensity categories: low, medium, high, or very high; breakdown plotted in **E**. (**F** and **G**) Random blood glucose at the beginning and end of treatment with either PBS or JNK inhibitor, expressed as absolute (**F**) or percentage change in blood glucose (**G**); (*n* ≥ 8). (**H**–**J**) Pancreas sections labeled for insulin (red), TUNEL (green), and DAPI (blue) were imaged (**H**) and quantified for the percentage of β cell labeling for TUNEL (**I**; *n* ≥ 8) or for β cell mass (**J**; *n* ≥ 8) at the end of the JNK inhibitor experiment. Males and females did not show differences for these outcomes and were combined. Scale bars: 50 μm. Statistics by 1-way (**D**, **G**, **I**, and **J**) or 2-way (**F**) ANOVA. *P < 0.05; **P < 0.01; ***P < 0.001; ****P < 0.0001.

Identification of IRE1-JNK-p53 as a driver of β cell death during GRP78 depletion suggested the possibility of therapeutic intervention to rescue β cells. First, we tested whether the ex vivo finding of JNK activation in islet cells after GRP78 knockdown reflected the biology of in vivo GRP78 deletion. Immunostaining pancreas sections from *Grp78^fl/fl^Ins1cre^+/–^* mice, with blinded quantitation of staining intensity, confirmed an increase in p-JNK abundance in GRP78-deleted mice relative to cre controls (Figure 7, A and B). Interestingly, non–β islet and exocrine cells did not show evidence of p-JNK labeling.

To test whether in vivo JNK inhibition could reduce β cell death in GRP78-deleted mice, we used a cell permeable, water-soluble JNK inhibitory peptide previously used for in vivo studies (32, 33). Injecting JNK inhibitory peptide once daily for 5 days on postnatal days (P) 8–12 (Figure 7C) resulted in a modest reduction in p-JNK staining intensity, decreasing the fraction of islets with the highest 2 categories of p-JNK intensity from 47% to 22% (Figure 7, D and E). This suggested partial efficacy of the systemically delivered inhibitor to reduce JNK activation in β cells in situ in the pancreas. Although we had designed the experiment to be preventative, starting treatment before the onset of hyperglycemia, these β cell Grp78-depleted pups proved to be already hyperglycemic at P8 (Figure 7F). Modulating diabetes trajectory after hyperglycemia is already established is a higher bar than prevention studies. Blood glucose in JNK inhibitory peptide-treated mice did not further increase, but was not restored to normal (Figure 7, F and G). Excitingly, pancreas sections from JNK inhibitory peptide-treated *Grp78*-deletion mice showed reduced β cell death at the end of the 5-day treatment compared with vehicle controls (Figure 7, H and I), although over this short time frame this was not sufficient to restore, or even partially increase, β cell mass (Figure 7J). Overall, the results suggested that short-term partial reduction of JNK signaling in β cells in vivo under ER stress or overactive UPR conditions can reduce β cell death even after hyperglycemia is already established.

### In vivo JNK inhibition reduces β cell death in Akita mice.

**Figure 8:**
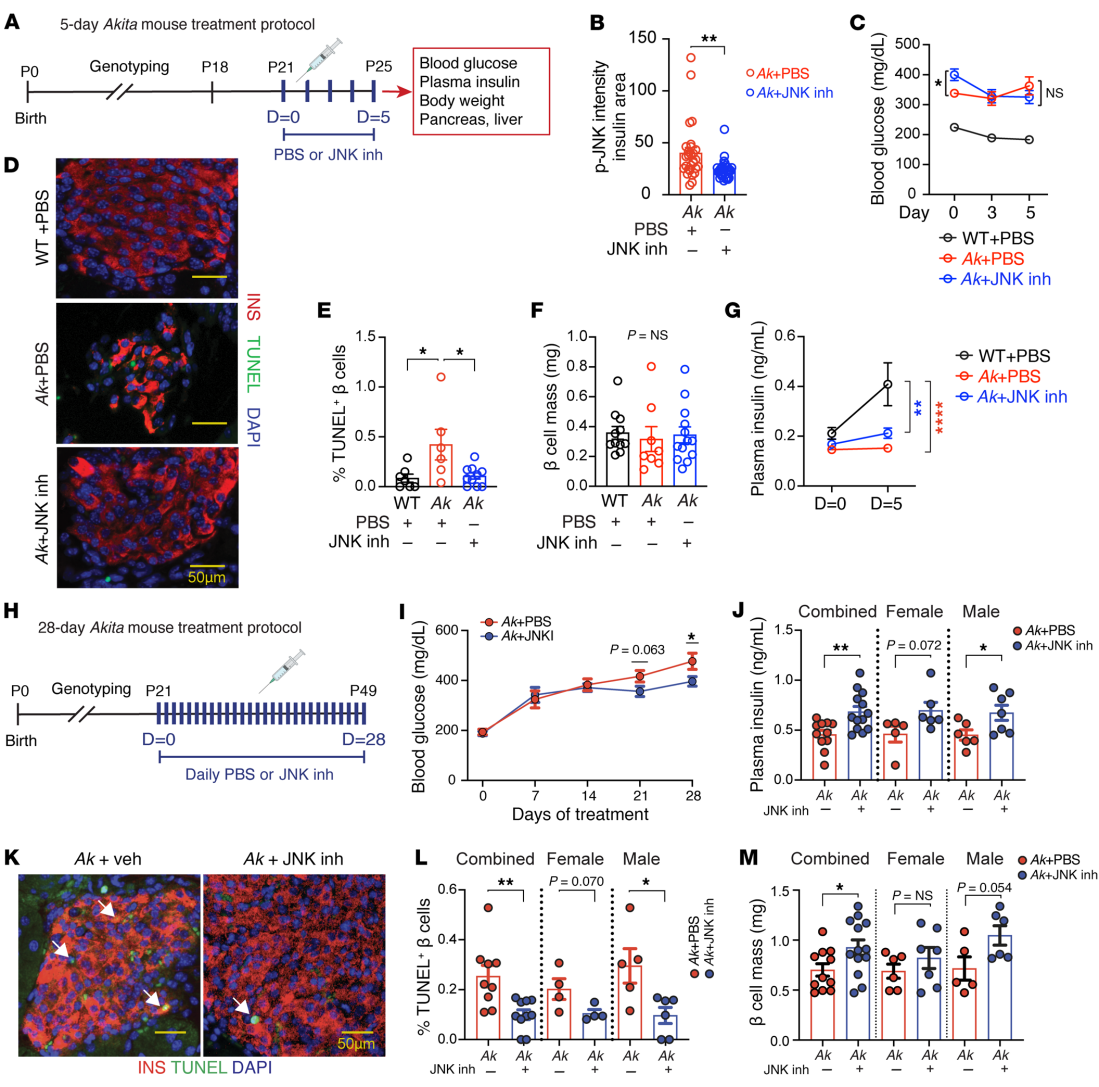
JNK inhibition decreases β cell death in Akita mice. (**A**) Schematic of 5-day in vivo treatment timeline and sample collection. (**B**) p-JNK staining intensity in insulin^+^ area of pancreas sections from Akita (Ak) mice with and without JNK inhibition; at least 8 islets were quantified from *n* = 3 mice in each group. (**C**) Random blood glucose at the beginning, day 3 and day 5 of treatment with either PBS or JNK inhibitor (*n* ≥ 15). (**D**–**G**) Pancreas sections (*n* ≥ 6) labeled for insulin (red), TUNEL (green), and DAPI (blue) were imaged (**D**) and quantified (**E**) for the percentage β cells that were TUNEL^+^. (**F**) β cell mass. (**G**) Plasma insulin levels at day 0 and day 5. (**H**) Schematic of 28-day in vivo treatment timeline. (**I**) Random blood glucose across the treatment period (*n* ≥ 11). (**J**) Plasma insulin levels (*n* ≥ 5). (**K**–**M**) Pancreas sections labeled for insulin (red), TUNEL (green) and DAPI (blue) were imaged (**K**) and quantified for the percentage β cells that were TUNEL^+^ (**L**) or for β cell mass (**M**). Males and females did not show marked differences for most outcomes and were combined for most panels. Scale bars: 50μm. Statistics by *t* test (**B** and **J**–**M**), 1-way ANOVA (**E**, **F**, **I**), or 2-way ANOVA (**C** and **G**). *P < 0.05; **P < 0.01; ***P < 0.001; ****P < 0.0001.

Since GRP78 deletion causes ER stress and general activation of all 3 UPR pathways, we wondered whether JNK inhibition might also protect β cells subjected to ER stress caused by other insults. The Akita *Ins2^C96Y^* A-chain mutation results in proinsulin misfolding leading to β cell failure due to ER stress and early diabetes (9, 34, 35). To test whether systemic JNK inhibition could protect β cells in this model, we injected Akita mice with JNK inhibitory peptide daily for 5 days starting at P21, taking blood samples for glucose and insulin measurements at baseline, day 3, and day 5, and then collecting tissues for molecular analyses and histology (Figure 8A). Pancreas sections immunostained for insulin, p-JNK, and DAPI confirmed p-JNK intensity was reduced in islets of JNK inhibitor–treated Akita mice relative to vehicle-treated Akita mice (Figure 8B). Three cJUN target genes reported to increase in diabetic liver (33, 36, 37), *Nfatc2*, *Pvr*, and *Txnip*, and 2 genes reported to be suppressed by cJUN, *Map1Lc3b*, *Lgals3,* were reversed in the Akita liver following JNK inhibition (Supplemental Figure 10), suggesting systemic activity of the JNK inhibitor.

To test whether JNK inhibition had antidiabetic activity in Akita mice, we measured blood glucose before and after treatment (Figure 8C). Despite randomly assigning the mice to treatment groups, the JNK inhibitor mice unfortunately started with higher mean blood glucose than the vehicle mice. However, over the 5-day treatment period, vehicle-injected mice had a slight worsening in blood glucose, whereas JNK inhibitor–injected mice had a slight improvement in blood glucose. We next examined whether JNK inhibition reduced β cell death in Akita mice. TUNEL staining showed that Akita mice had increased β cell death compared with littermate controls (Figure 8, D and E). Excitingly, JNK inhibition reduced β cell death in Akita mice back to the level observed in controls, although β cell mass was not impacted over this short-term intervention (Figure 8F). Wild-type littermate controls had a marked increase in circulating insulin over the 5-day trial (Figure 8G), possibly related to the study being performed around the time that mice transition from fat-rich milk to carbohydrate-rich chow (38). Akita mice did not show a corresponding increase, although circulating random insulin levels were slightly improved by JNK inhibition. To test whether a longer period of JNK inhibition could reduce β cell death and increase β cell mass, we injected Akita mice with JNK inhibitor daily for 28 days starting at 3 weeks of age (Figure 8H). Blood glucose was only subtly improved by JNK inhibition, possibly due to intrinsic impairment in insulin production, secretion, and bioactivity related to the Akita *Ins2^C96Y^* mutation (Figure 8I). Circulating immunoreactive insulin only slightly increased over this time frame as well (Figure 8J). Similar to the 5-day treatment the proportion of β cell labeling for TUNEL was reduced by JNK inhibition (Figure 8, K and L), and after 28 days’ treatment, this translated to an increase in β cell mass as well (Figure 8M).

### Depleting GRP78 in human islets causes human β cell death via JNK and p53.

**Figure 9:**
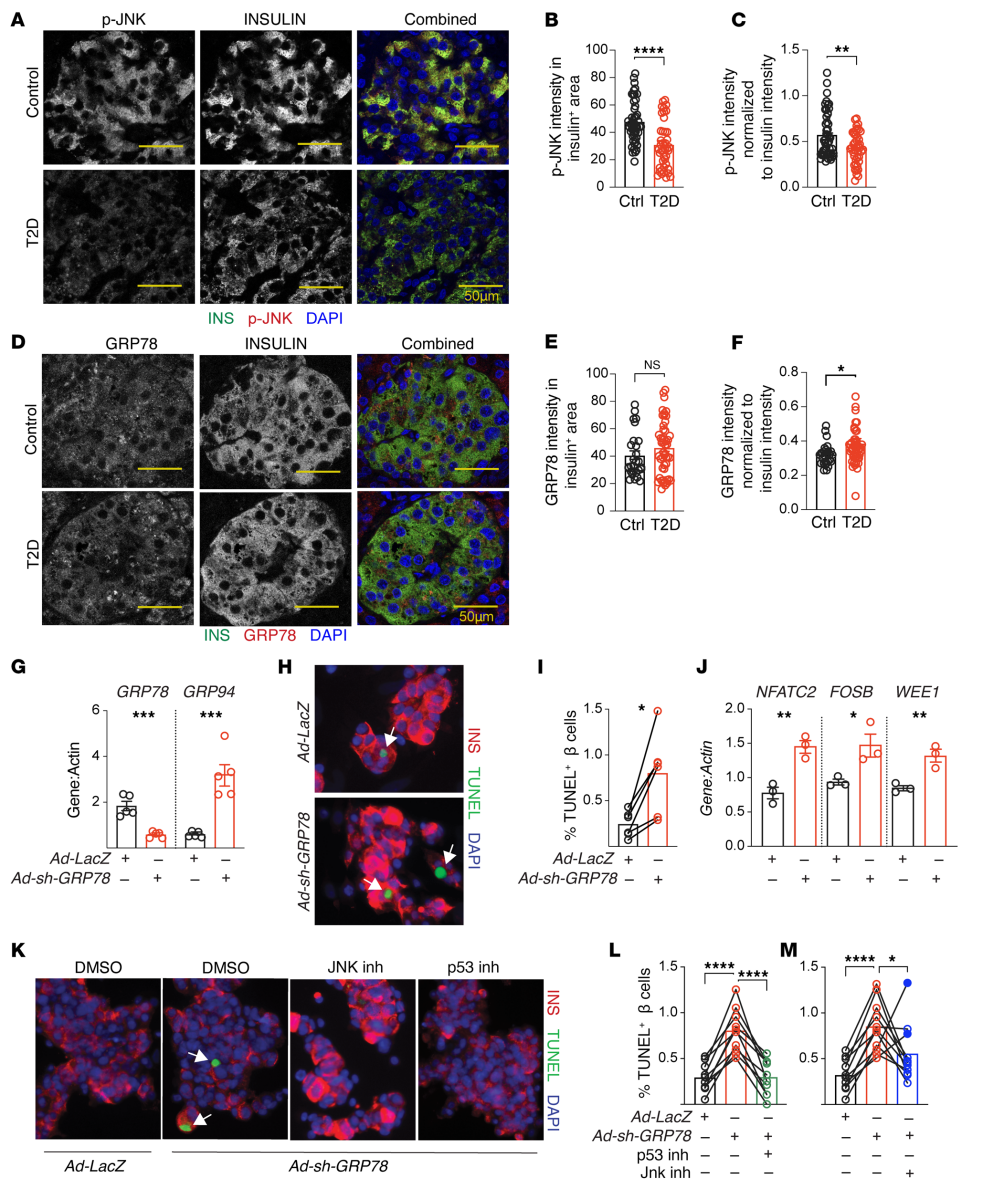
Inhibition of either JNK or p53 reduces human β cell death after GRP78 depletion. Panels **A**–**F** contain data from human pancreatic sections, and panels **G**–**M** contain data from ex vivo culture of dispersed human islet cells transduced with *Ad-LacZ* or *Ad-sh-GRP78* and cultured for 72 hours. (**A**–**C**) Sections from individuals with T2D or controls (*n =* 5 of each) were immunostained for insulin (green), p-JNK (red), and DAPI (blue), imaged with confocal microscopy (**A**), and p-JNK staining intensity over the insulin^+^ area quantified using ImageJ either in absolute (**B**) or normalized to insulin staining intensity (**C**). (**D**–**F**) Sections from individuals with T2D or controls (*n =* 5 each) were immunostained for insulin (green), GRP78 (red), and DAPI (blue), imaged with confocal microscopy (**D**), and GRP78 staining intensity over the insulin^+^ area quantified using ImageJ either in absolute (**E**) or normalized to insulin staining intensity (**F**). (**G**) Dispersed human islet cells transduced with *Ad-LacZ* or *Ad-sh-GRP78* were assessed for *GRP78* or *GRP94* abundance by qPCR (*n* = 5). (**H** and **I**) Dispersed human islet cells transduced with *Ad-LacZ* or *Ad-sh-GRP78* and plated on glass coverslips (*n* = 6) were assessed for apoptosis by labeling for insulin (red), TUNEL (green), and DAPI (blue); imaged (**H**); and quantified for the percentage of β cells labeling for TUNEL (**I**). (**J**) Human islet cells treated as in **G** were assessed by qPCR for abundance of cJUN target genes (*n* = 3). (**K**–**M**) Dispersed human islet cells transduced with *Ad-LacZ* or *Ad-sh-GRP78* were treated with vehicle (DMSO), JNK-IN-8 inhibitor (*n* = 11), or p53 inhibitor (*n* = 10) for 72 hours; labeled for insulin (red), TUNEL (green), and DAPI (blue); imaged (**K**); and quantified for the percentage of β cells labeling for TUNEL (**L** and **M**). Statistics by *t* test (**B**, **C**, **E**–**G**, **I**, and **J**) or 1-way ANOVA (**L** and **M**). *P < 0.05; **P < 0.01; ***P < 0.001; ****P < 0.0001.

β cells from individuals with T2D show evidence of ER stress (39); however, to our knowledge, it is unknown whether JNK is activated in human β cells in situ in the pancreas during T2D. We obtained pancreas sections from 10 individuals: 5 with T2D and 5 controls matched for sex and BMI. Immunostaining for p-JNK and insulin (Figure 9A) showed that, contrary to our hypothesis, p-JNK was less abundant in insulin-positive areas in these individuals with T2D compared with individuals who were controls, even when normalized to insulin intensity (Figure 9, B and C). Since our initial mouse observations were performed after forced reduction of GRP78, and T2D has been associated with increased GRP78 expression (e.g., as in ref 40), we assessed GRP78 abundance in these samples. GRP78 protein abundance was not increased in islets in these T2D sections but was slightly increased when normalized to insulin staining intensity (Figure 9, D–F). Given this result, we wondered whether p-JNK abundance was in fact increased in the Akita mouse model in which we had previously observed metabolic improvement with JNK inhibition (Figure 8), since Akita is also known to have increased GRP78 expression (41). Interestingly, the Akita mouse pancreas also showed a lack of increase in p-JNK staining intensity compared with controls (data not shown). As such, we conclude that, although diabetogenic JNK activity is present, as evidenced by cJUN targets increased and cell death improvement after JNK inhibition, diabetogenic JNK activity in β cells may not correlate with islet p-JNK staining intensity on paraffin sections.

To test whether reducing GRP78 negatively impacts human β cell survival, we transduced dispersed human islet cells with adenovirus expressing shRNA targeting *GRP78*. *Ad-sh-GRP78* achieved a 70%–80% reduction of *GRP78* mRNA at 72 hours after transduction, along with compensatory induction of *GRP94*, a functional readout for loss of GRP78 protein (Figure 9G). *GRP78* knockdown increased human β cell apoptosis, measured by TUNEL staining, in all donor preparations tested (Figure 9, H and I). Similar to ex vivo mouse findings, IRE1 inhibition after GRP78 knockdown in human islets rescued β cell death, confirming that IRE1 activation contributes to human β cell apoptosis (Supplemental Figure 11). *GRP78* knockdown increased the expression of cJUN target genes *NFATC2*, *FOSB*, and *WEE1*, suggesting that the reduction of GRP78 activates JNK in human islet cultures as it does in mice (Figure 9J). To test whether JNK, p53, or both participate in human β cell apoptosis after GRP78 is knocked down, we performed the *Ad-sh-GRP78* knockdown experiment on dispersed human islet cell cultures with and without JNK inhibitor or p53 inhibitor. TUNEL staining of these cultures (Figure 9K) showed that p53 inhibition (Figure 9L) and JNK inhibition (Figure 9M) rescued β cell death in most samples. Taken together, these data suggest that inhibiting IRE1, JNK, or p53 can rescue human β cells from ER stress–related β cell death in the setting of GRP78 reduction.

## Discussion

β cell loss due to apoptosis is an important cause of insulin deficiency in diabetes pathogenesis. This study sheds light on the molecular mechanisms leading to β cell death under ER stress conditions, relevant to both T1D and T2D (5, 6). After deleting the key ER chaperone GRP78, we observed that β cell death was not due to activation of CHOP, but rather to IRE1 kinase activity leading to JNK phosphorylation and p53 activation. Excitingly, systemic JNK inhibition was protective against β cell death in 2 live-animal β cell–specific models of ER stress–induced diabetes, and longer-term JNK inhibition measurably preserved β cell mass. Both JNK inhibition and acute p53 inhibition reduced ER stress–induced apoptosis in human β cells. These studies add to our understanding of mechanisms of β cell death during ER stress and outline possible therapeutic approaches for intervention.

Our study sheds light on whether β cell death during ER stress conditions is due to unmitigated stress versus hyperactivation of the cellular response to stress. GRP78 is a critical ER chaperone; deleting GRP78 increases cellular stress (42). GRP78 also acts as a suppressive gatekeeper for the UPR (5). Molecular studies confirmed activation of all 3 UPR pathways after GRP78 deletion in mouse islet cells; inhibition of IRE1 restored cell death levels back to baseline. Inhibition of PERK also reduced cell death, although the result did not achieve statistical significance. Surprisingly, CHOP reduction did not rescue cell death, suggesting an alternate mechanism downstream of PERK (7). ATF6 inhibition, on the other hand, increased cell death and decreased cell number, even in the absence of GRP78 knockdown, consistent with prior reports that ATF6 exerts prosurvival effects on β cells. Taken together, these results suggest that UPR hyperactivation via IRE1 and PERK was the proximate cause of cell death in this model rather than the toxicity of ER stress itself.

Our data support IRE1 as a therapeutic target in β cell preservation. β cell toxicity caused by IRE1 hyperactivation results from Regulated IRE1 Dependent Decay–mediated (RIDD-mediated) mRNA degradation (8) and from IRE1 kinase activity (24). JNK inhibition effectively reduced cell death in the models tested in this study, suggesting IRE1 kinase activity played an important role. IRE1 kinase phosphorylates JNK via TRAF2 (24). IRE1 nuclease inhibitors are under investigation as therapeutic options for diabetes (43, 44); separately, β cell IRE1 reduction protected against T1D through a dedifferentiation mechanism (45). Although IRE1 plays important homeostatic roles in adaptation and function in many cell types that could limit the safety of IRE1 inhibitors, agents that target hyperactivation while leaving basal activation intact may be possible (8).

Therapeutic inhibition of one or more JNK isoforms may improve β cell survival. Pan-isoform JNK inhibition reduced murine β cell death both ex vivo and in 2 different in vivo models, including the relatively severe Akita β cell ER stress model caused by misfolded proinsulin. Although JNK inhibition was not sufficient to reverse diabetes, both 5- and 28-day treatment reduced β cell death, and 28-day treatment improved β cell mass. Neither treatment paradigm fully rescued blood glucose or plasma insulin levels, possibly related to upstream negative impact of the Akita mutation, which impacts bystander healthy proinsulin molecules as well (46). Importantly, JNK inhibition also protected human β cells from apoptosis in our ex vivo culture model. We considered whether an increase in β cell proliferation might have contributed to the β cell mass increase with JNK inhibition, but this seems unlikely, given the downregulation of proliferation-related gene sets like E2F targets, MTORC1 signaling, and Glycolysis in the RNA-seq dataset (Figure 6A). JNK kinases, of which there are 3 isoforms, have important roles in tissues throughout the body, including as a negative regulator of insulin signaling (47). JNK depletion or inhibition has been previously shown to reduce β cell death in cell culture stress models (32, 48–50), in vivo in mice (51–53), and in human islet engraftment models (54–57). However, pan-JNK inhibition worsened albuminuria in mice, a sign of kidney toxicity (58). JNK biology is complicated and requires more investigation before a therapeutic can be safely advanced. For example, JNK3, expressed in β cells, may have protective roles (59, 60). On the other hand, JNK3 plays important roles in neurological development and function, yet JNK3 inhibitors are under exploration for neuroprotective therapy (61). Further work is required to identify safe and efficacious approaches to target JNK isoforms for β cell preservation.

Our data suggest that p53 acts downstream of JNK to cause β cell death during ER stress. JNK is a known activator (26, 27) or suppressor (62) of p53 activity in other cell types and cancers. In islet cells, we observed a p53 transcriptional signature when GRP78 was deleted, and the signature was reduced by JNK inhibition. Furthermore, JNK inhibition reduced phospho-p53 labeling of β cell nuclei and reduced p53 target gene expression by qPCR, and p53 inhibition reduced β cell death in mouse and human islet cell cultures. These results warrant cautious excitement. We have found only 2 prior publications implicating a similar JNK-p53 pathway in β cell death, one after cytokine exposure (63) and one after FFA exposure (64); both were primarily in transformed cell lines and provide no data in human β cells. Like TUNEL, only a small fraction of β cell nuclei labeled for phospho-p53, suggesting this is a fleeting stage that precedes cell death execution. Islet p53 activation was previously observed in mouse T2D models (29) and in human pancreatic β cells from T2D sections (31); p53 activity was associated with dsDNA breaks in a glucotoxicity model (31). Whether p53 inhibition improves β cell survival under stress conditions has been an unresolved question, however. Chronic β cell–specific p53 deletion in mice did not protect against loss of β cell mass after STZ, despite a reduction in pro-apoptotic mRNAs (30). Our results suggest that acute p53 inhibition, either pharmacologic or genetic, substantially prevented β cell death during UPR activation, suggesting that effects may differ between acute and chronic inhibition. Acute p53 inhibition may avoid compensatory changes that blunt the survival benefit.

This study provides insight into the roles of GRP78 in mouse and human β cells. β cells express GRP78 under basal conditions and increase GRP78 abundance during stress (3). Loss of GRP78 cochaperones DNAJC3, ERDJ4, or its nucleotide exchange factor SIL1 causes diabetes (65–69), and proinsulin is a GRP78 folding client (11, 70–72). Forced overexpression of GRP78 in β cells imparted resistance to metabolic decompensation during high-fat feeding (73), and knockdown of *Grp78* worsened lipotoxic cell death in a transformed β cell line (74). Heterozygous GRP78 loss of function in all tissues, which resulted in 50% reduction in GRP78 protein level, caused resistance to weight gain during high-fat feeding and reduced insulin levels in the context of improved insulin sensitivity (75). Deletion of GRP78 from all pancreatic cell types using *Pdx1-cre* (76) led to disruption of the exocrine compartment, with most acinar cells replaced by fat. Glucose intolerance occurred, but islet studies were limited by the severe exocrine pathology and by the histochemical observation that remaining β cells appeared to have escaped GRP78 reduction. In the current study, GRP78 proved essential for both mouse and human β cell survival, even under basal unstressed conditions. Heterozygous deletion of GRP78 was innocuous, suggesting that β cells can tolerate a partial reduction in GRP78.

The study has several limitations. The RNA-seq experiment was performed on bulk material rather than single cells, and was performed on islet cultures after ex vivo *Grp78* knockdown rather than islets obtained from the in vivo environment. The ex vivo knockdown paradigm used adenovirus reagents, which could introduce additional stress; multiplicity of infection was matched across all conditions. The in vivo JNK inhibitor experiments proved insufficient to normalize blood glucose in Akita mice, despite reducing ongoing β cell death and improving β cell mass. Approaches to improve β cell survival may not be sufficient to prevent or reverse diabetes if additional defects besides cell death are present, such as failure of insulin production or secretion.

In sum, with this study we report that GRP78 suppression of UPR activation is essential for β cell survival, without which diabetes rapidly ensues. Molecular studies identify the mechanism driving cell death as IRE1 kinase–dependent JNK activation leading to p53-mediated cell death. Therapeutic studies show that inhibiting either JNK or p53 rescues mouse and human β cell death, and in vivo JNK inhibition reduced β cell apoptosis in diabetes. Taken together, this work identifies a path to β cell death and identifies possible approaches to intervene.

## Methods

### Sex as a biological variable.

For in vivo and in vitro studies in mice, both sexes were used, and data were analyzed together without any bias. Similarly, human pancreas sections and islets from both sexes were obtained from the Integrated Islet Distribution Program (IIDP) and analyzed together.

### Mouse models.

*Grp78^fl/fl^* mice were a kind gift from Dr. Amy S. Lee (21) and are now available at Jackson laboratories (*B6.129(Cg)-Hspa5^tm1.1Alee^*/J; RRID:IMSR_JAX:019549). To generate β cell-specific *Grp78* knockdown, we crossed *Grp78^fl/fl^* mice with mice harboring cre-recombinase at the *Ins1* gene locus (*B6(Cg)-Ins1^tm1.1(cre)Thor^*/J; RRID:IMSR_JAX:026801) (22). Some studies were performed in *Akita* mice (*C57BL/6-Ins2^Akita^*/J; RRID:IMSR_JAX:003548) or *mTmG* mice (*Gt(ROSA)26Sor^tm4(ACTB–tdTomato,–EGFP)Luo^*/J; RRID:IMSR_JAX:007576. All mice were housed in a temperature-controlled room with 12-h light/dark cycle and continuous access to standard mouse chow and water. Both male and female mice were used for all experiments, at the ages described in the text and figures. For BrdU labeling, mice were injected with 10 mg/mL bromodeoxyuridine twice intraperitoneally (IP), 4 and 2 hours before tissue harvest (35). Tail blood was collected for measuring blood glucose, plasma insulin, and glucagon. For in vivo JNK inhibition, JNK inhibitory peptide D-JNKI-1 (Med chem Express) was injected IP once a day (0.5 mg/kg) starting at day 8 for Ins1 Cre Grp78 mice and at day 21–23 for AKITA mice for 5 consecutive days in PBS. *Grp78^fl/fl^* male and female mice at 12–60 weeks of age were used for ex vivo islet studies.

### Mouse islet isolation and cell culture.

General reagents and sources are listed in Supplemental Table 1. Islets were isolated by injecting collagenase in the common bile duct, followed by separating endocrine and exocrine tissue by Ficoll-Histopaque density gradient as described previously (35). After resting overnight, islets were dispersed to single cells using 0.05% trypsin, plated on plastic for RNA and protein or on glass coverslips for immunostaining and microscopy, and cultured in islet complete media (ICM) containing RPMI 1640 with no glutamine and no glucose, 10% FBS, penicillin/streptomycin and supplemented with 15 mM glucose in 24-well plates in a 37°C humidified incubator with 5% CO_2_. Other additives are added at the start of the experiment, as described below. Adenovirus (Supplemental Table 2) expressing *LacZ* (control) or *Cre* recombinase (knockdown) were used at a multiplicity of infection (MOI) of 20 to knock down *Grp78* in parallel on *Grp78^fl/fl^* islet cells from the same biological replicate. Islet cells were harvested for molecular studies 72 hours after glucose and adenoviral treatment. All experimental conditions were matched for total virus MOI. Culture additives (Supplemental Table 3) included: Atf6 inhibitor (AEBSF, Thermo Fisher Scientific), PERK Inhibitor (GSK2606414, Med Chem Express), IRE1 Inhibitor (4μ8c, Selleck Chem), IRE1 kinase inhibitor (APY29, Med Chem Express), IRE1 nuclease inhibitor (SPY-083010, Calbiochem), JNK inhibitor (JNK-IN-8, Selleck Chem), and p53 inhibitor (PFT-α, Selleck Chem). All the inhibitors are added at the start of the experiment for 72 hours.

### Human islet cell cultures.

Human cadaveric islets (Supplemental Table 4) were received from IIDP in CMRL (Connaught Medical Research Laboratories) 1066 based media on ice. After receiving, human islets were centrifuged at 1500rpm for 5 min at RT and the shipping media was discarded. The human islet pellet was resuspended in RPMI-based ICM containing 10% FBS, penicillin/streptomycin, supplemented with 5 mM glucose, and transferred in 10 cm tissue culture plates with minimum binding. Human islets were allowed to recover from the shipping and handling stress for 24 hours by incubating them in a 37°C humidified incubator with 5% CO_2_. Human islets were dispersed using 0.05% trypsin described previously. Islet cells were either plated on plastic (100 IEQs) for RNA/Protein isolation or on glass coverslips (50 IEQs) for performing immunostaining. All human islet cell experiments were performed in 15 mM glucose for 96 hours. At the start of the experiment, dispersed human islet cells were transduced with adenoviruses expressing β-galactosidase (*Ad-LacZ*) or human *GRP78* short hairpin RNA (*Ad-sh-GRP78*) were used at an MOI of 20 to knockdown GRP78, and the chemical inhibitor were also added at the same concentration as described above in the mouse cell culture section. At the end of the treatment, cells were fixed and stained, as described below.

### Human pancreas sections.

Human pancreas sections (Supplemental Table 5) were received from the Integrated Islet Distribution Program (IIDP). p-JNK and insulin immunofluorescence staining was performed on five non-diabetic and five T2D pancreas sections similar to the mouse pancreas sections described below.

### Histology and immunofluorescence.

Mouse pancreas tissue and duodenum were dissected and fixed for 5 hours (4 hours for mTmG pancreata) with 10% formalin at room temperature. After fixation, tissues were processed, paraffin embedded, and sectioned (5 μm) at either the UMASS Medical School or Weill Cornell Medicine histopathology cores. Islet cell cultures were fixed in 4% paraformaldehyde for 10 min at room temperature. For BrdU immunofluorescence, fixed cells or rehydrated paraffin sections were submerged in 1 N HCl for 25–30 min at 37°C, blocked for 2 hours in goat serum–based block with 0.1% Tween 20, labeled with primary antibodies, then secondary antibodies (Invitrogen), and mounted on glass slides with Fluoroshield mounting media containing DAPI (Sigma-Aldrich). For TUNEL, CS and IHC sections were stained according to the manufacturers protocol (Promega) and after end labelling, cells were counterstained with insulin, glucagon and DAPI as previously described (35). For insulin and glucagon IHC, sections were stained as previously described (77). Immunostaining for p-JNK and p-p53 was performed by incubating the paraffin sections with 0.1% triton for 10 min followed by insulin immunostaining protocol. The primary antibodies (Supplemental Table 6) were guinea pig anti-insulin (catalog #A0564; Dako), anti-glucagon (catalog #2760; cell signaling), anti-somatostatin (catalog #30788; Abcam), rabbit anti-pJNK (catalog #4668; Cell Signaling Technology), mouse anti-p-p53 (catalog #9284; Cell Signaling Technology), rat anti-BrdU (catalog Ab6326; Abcam). Fluorescence images were acquired using a Nikon TE2000-E2 inverted microscope or TissueGnostics NIKON microscope. Brightfield images at 200X were acquired of islets from pancreas sections stained for insulin (DAB) and hematoxylin using on Nikon TE2000-E2 inverted microscope. Whole pancreas sections were also scanned on slide scanner for α or β cell mass, which was estimated as the product of the percent glucagon- or insulin-labeled area, quantified using Adobe Photoshop and Image J, and the pancreas weight.

### Confocal microscopy.

Dispersed human islets cultured on glass coverslips transduced with adenoviruses and treated with inhibitors as described above were fixed using 4% paraformaldehyde for 10 min at room temperature. Human islet cells and human pancreas sections were immune stained for Insulin, p-p53, p-JNK, TUNEL, and DAPI, as described in the previous section. Both human islet cells and human pancreas sections were imaged using a Zeiss LSM 880 inverted confocal microscope. All the images were acquired using 63X objective. We used the same exposure time for each laser to capture the immunostaining of human islet cells from the same experiment with different treatments and similarly for all the human pancreas sections.

### Flow cytometry.

Dispersed islet cells from 50–100 islet equivalents (IEQ) were cultured on 24-well tissue culture treated plastic plates as above, then were detached from the plate using 0.05% trypsin as previously described (35). Cells were washed twice with ice-cold PBS and suspended in 1X buffer for staining with Annexin V/PI per the manufacturer’s protocol (Millipore). Cells were washed twice with ice-cold PBS and kept on ice until flow cytometric counting with BD Accuri C6 flow cytometer (BD Biosciences). All the samples counted a minimum of 15,000 cells. Experiments were analyzed using FlowJo software.

### Gene expression.

For RNA, islet cells after culture were washed with ice-cold PBS, or liver, spleen, and muscle were suspended/homogenized in SKP buffer supplemented with 10% β-mercaptoethanol from Norgen RNA/Protein isolation kit (Norgen, Canada). Total RNA was isolated from cells/tissues using the manufacturer’s protocol. 250–1,000 ng of total RNA underwent cDNA synthesis using Superscript IV VILO kit from Invitrogen. Gene expression was measured by SYBR green QPCR using primers listed in Supplemental Table 7 (mouse) and Supplemental Table 8 (human). Data are expressed as ddCT (fold change) normalized to actin.

### Western blots.

Total protein was extracted from islet cell cultures using the protein lysis buffer described previously (77) or isolated from RNA/protein spin columns from the Norgen kit (Canada). Samples prepared using protein lysis buffer were subjected to sonication (QSONICA) for 10 min on 4°C with 30-sec pulse and 30-sec rest. After centrifugation at 12000 *rpm* for 5 min at 4°C, reducing SDS-PAGE sample buffer (Invitrogen) was added and the samples were boiled for 10 min. Samples were allowed to cool down at room temperature and subjected to separation on 4–20% precast gels (Bio-rad) for 1 hour at 140 volts. Separated proteins were transferred to PVDF membrane for 1 hour 15 minutes at 100 volts in a 4°C cold room. Membranes were blocked for 1 hour with either 10% skim milk powder or 5% bovine serum albumin (BSA) to minimize nonspecific antibody binding to the membrane. The membranes were incubated with primary antibodies (Supplemental Table 6) diluted in 2.5% BSA overnight. After washing the membranes three times (1XPBST), a species-specific secondary HRP-conjugated antibody was added at 1:2000 dilution for 1 hour. Blots were developed using ECL, ECL prime (GE Healthcare), or SuperSignal West Femto Maximum Sensitivity Substrate (Thermo Scientific) on autoradiography films or Bio-rad gel imager. Western blot bands were quantitated using ImageJ. Densitometric band quantification data were internally normalized to remove bias due to difference in background. The densitometric data were presented as protein normalized to actin.

### Bulk RNA sequencing.

Islet cell cultures from 2 male and 2 female *Grp78^fl/fl^* mice transduced with *Ad-LacZ* or *Ad-cre* and treated with vehicle or JNK inhibitor for 72 hours were harvested for RNA isolation as described above. RNA integrity number for most samples was above 9.2. 250 μg of total RNA was used for polyA library preparation and paired-end PE150 RNA sequencing on an Illumina HiSeq 4000 (Quick Biology, USA). The data were received in fasq files that were aligned to the mouse genome to generate raw counts (78). 17–25 million paired-end raw counts were obtained from each sample. Differently expressed genes between samples were analyzed using UMASS Chan Medical School free software DEBrowser (79). Gene counts were filtered for genes with >1 count per million (CPM) for analysis, which was then normalized using the trimmed mean of M-values (TMM) method with *P* < 0.05 and FC>1.5 to generate DE gene lists. Also, normalized all detected gene lists were generated using DEBrowser software, which was further analyzed with free online software GSEA (Broad Institute and UC San Diego) using a Hallmark gene enrichment data set to generate differently expressed pathways with FDR less than 25% or TRRUST transcription factor analysis was performed using free online resource Enrichr (80).

### Immunofluorescence image quantification.

Image data were quantified in an unbiased manner by blinding the images and then quantifying using Cell Profiler automated counting software from the Broad Institute (80) or Strataquest (TissueGnostics, USA). At least two images were manually checked post-quantification to confirm the data and assess for errors in the automated counts. TUNEL, p-p53 and BrdU positive β cells were manually assessed by blinding the IHC and CS images to remove any bias which were confirmed by two different individuals in the lab. 1000–2000 β cells were counted for each experimental condition. ImageJ was used to estimate p-JNK intensity in the insulin positive area for mouse and human pancreas sections. For IHC sections, 5–10 islets were imaged from each mouse or human pancreatic sections, which were analyzed either manually for p-JNK, BrdU, p-p53 and TUNEL or automation was performed for insulin and glucagon cell counts as mentioned above.

### Institutional cores.

We used the morphology and histology core facility at UMASS Chan Medical School and Laboratory of Comparative Pathology, Center of Comparative Medicine and Pathology, Sloan Kettering Institute, to process, embed, and cut 5-micron pancreas sections. The paraffin sections were immunostained with insulin in our lab and counter-staining for hematoxilin was performed at the institutional cores. We also used the Weill Cornell Medicine Microscopy and Image Analysis Core Facility for confocal imaging using a Zeiss LSM 880 inverted microscope.

### Statistics.

All the data are presented as mean ± SEM. The Statistical analyses were performed using Prism 10. Statistical significance was calculated by 2-tailed Student’s *t* test for comparing 2 groups or 1-way ANOVA for multiple comparisons; *P* < 0.05 was considered significant. The number of replicates and the statistical test used for each experiment are described in the figure legends.

### Study approval.

All mouse procedures were approved by the Institutional Animal Care and Use Committees at UMass Medical School and Weill Cornell Medicine.

### Data availability.

All reagents used in the study are available commercially. Source details are provided in the Supplemental Tables. RNA-seq data have been deposited at GEO (accession number GSE282680) and are publicly available as of the date of publication. All other data reported in this paper can be found in the Supporting Data Values file.

## Author contributions

LCA, JKK, and ASL initially conceptualized the study; LCA acquired funding. LCA and RBS conceptualized and designed the experiments. RBS, CD, YW, TDL, TAC, BG, and AR performed the studies. DR performed the initial bioinformatics data analysis. LCA and RBS analyzed the data and wrote the manuscript; all authors had the opportunity to review and approve the manuscript.

## Conflict of interest

ASL is a scientific advisory board member of BiPER Therapeutics. LCA has performed consulting work for Eli Lilly and Co., Sanofi S.A., and Vertex Pharmaceuticals Inc.

## Funding support

This work is the result of NIH funding, in whole or in part, and is subject to the NIH Public Access Policy. Through acceptance of this federal funding, the NIH has been given a right to make the work publicly available in PubMed Central.

- United States National Institutes of Health (R01DK113300, LCA; R01DK114686, LCA; R01DK135304, LCA; R01DK124906, LCA; and F31DK136225, AR).
- NIH Grant # U24DK098085.

## Supplementary Material

Supplemental data

Unedited blot and gel images

Supporting data values

## Version 1 08/03/2026

Electronic publication

## References

[1] Weir GC, Bonner-Weir S (2013). Islet β cell mass in diabetes and how it relates to function, birth, and death. Ann N Y Acad Sci.

[2] Arunagiri A (2019). Proinsulin misfolding is an early event in the progression to type 2 diabetes. Elife.

[3] Back SH, Kaufman RJ (2012). Endoplasmic reticulum stress and type 2 diabetes. Annu Rev Biochem.

[4] Sharma RB (2021). Living dangerously: protective and harmful ER stress responses in pancreatic β cells. Diabetes.

[5] Shrestha N (2021). Pathological β cell endoplasmic reticulum stress in type 2 diabetes: current evidence. Front Endocrinol (Lausanne).

[6] Tersey SA (2012). Islet β cell endoplasmic reticulum stress precedes the onset of type 1 diabetes in the nonobese diabetic mouse model. Diabetes.

[7] Song B (2008). Chop deletion reduces oxidative stress, improves beta cell function, and promotes cell survival in multiple mouse models of diabetes. J Clin Invest.

[8] Ghosh R (2014). Allosteric inhibition of the IRE1α RNase preserves cell viability and function during endoplasmic reticulum stress. Cell.

[9] Støy J (2007). Insulin gene mutations as a cause of permanent neonatal diabetes. Proc Natl Acad Sci U S A.

[10] Stone SI (2021). Monogenic and syndromic diabetes due to endoplasmic reticulum stress. J Diabetes Complications.

[11] Scheuner D (2005). Control of mRNA translation preserves endoplasmic reticulum function in beta cells and maintains glucose homeostasis. Nat Med.

[12] Abdulkarim B (2015). A missense mutation in PPP1R15B causes a syndrome including diabetes, short stature, and microcephaly. Diabetes.

[13] Lekszas C (2020). Biallelic TANGO1 mutations cause a novel syndromal disease due to hampered cellular collagen secretion. Elife.

[14] Montaser H (2021). Loss of MANF causes childhood-onset syndromic diabetes due to increased endoplasmic reticulum stress. Diabetes.

[15] Delépine M (2000). EIF2AK3, encoding translation initiation factor 2-alpha kinase 3, is mutated in patients with Wolcott-Rallison syndrome. Nat Genet.

[16] Yang J (2022). IER3IP1 is critical for maintaining glucose homeostasis through regulating the endoplasmic reticulum function and survival of β cells. Proc Natl Acad Sci U S A.

[17] Yong J (2021). Therapeutic opportunities for pancreatic β cell ER stress in diabetes mellitus. Nat Rev Endocrinol.

[18] Shiu RP (1977). Glucose depletion accounts for the induction of two transformation-sensitive membrane proteinsin Rous sarcoma virus-transformed chick embryo fibroblasts. Proc Natl Acad Sci U S A.

[19] Haas IG, Wabl M (1983). Immunoglobulin heavy chain binding protein. Nature.

[20] Ni M, Lee AS (2007). ER chaperones in mammalian development and human diseases. FEBS Lett.

[21] Luo S (2006). GRP78/BiP is required for cell proliferation and protecting the inner cell mass from apoptosis during early mouse embryonic development. Mol Cell Biol.

[22] Thorens B (2015). Ins1(Cre) knock-in mice for beta cell-specific gene recombination. Diabetologia.

[23] Chen C-W (2022). Adaptation to chronic ER stress enforces pancreatic β cell plasticity. Nat Commun.

[24] Urano F (2000). Coupling of stress in the ER to activation of JNK protein kinases by transmembrane protein kinase IRE1. Science.

[25] Zhang T (2012). Discovery of potent and selective covalent inhibitors of JNK. Chem Biol.

[26] Fuchs SY (1998). MEKK1/JNK signaling stabilizes and activates p53. Proc Natl Acad Sci U S A.

[27] Gottifredi V, Prives C (2001). Molecular biology. Getting p53 out of the nucleus. Science.

[28] Vousden KH, Lane DP (2007). p53 in health and disease. Nat Rev Mol Cell Biol.

[29] Belgardt B-F (2015). The microRNA-200 family regulates pancreatic beta cell survival in type 2 diabetes. Nat Med.

[30] Uhlemeyer C (2023). Selective ablation of P53 in pancreatic beta cells fails to ameliorate glucose metabolism in genetic, dietary and pharmacological models of diabetes mellitus. Mol Metab.

[31] Tornovsky-Babeay S (2014). Type 2 diabetes and congenital hyperinsulinism cause DNA double-strand breaks and p53 activity in β cells. Cell Metab.

[32] Bonny C (2001). Cell-permeable peptide inhibitors of JNK: novel blockers of beta cell death. Diabetes.

[33] Kaneto H (2004). Possible novel therapy for diabetes with cell-permeable JNK-inhibitory peptide. Nat Med.

[34] Yoshioka M (1997). A novel locus, Mody4, distal to D7Mit189 on chromosome 7 determines early-onset NIDDM in nonobese C57BL/6 (Akita) mutant mice. Diabetes.

[35] Sharma RB (2015). Insulin demand regulates β cell number via the unfolded protein response. J Clin Invest.

[36] Hirosumi J (2002). A central role for JNK in obesity and insulin resistance. Nature.

[37] Schummer P (2016). Specific c-Jun target genes in malignant melanoma. Cancer Biol Ther.

[38] Burton JJ, Alonso LC (2024). Overnutrition in the early postnatal period influences lifetime metabolic risk: evidence for impact on pancreatic β cell mass and function. J Diabetes Investig.

[39] Christensen AA, Gannon M (2019). The beta cell in type 2 diabetes. Curr Diab Rep.

[40] Brusco N (2023). Intra-islet insulin synthesis defects are associated with endoplasmic reticulum stress and loss of beta cell identity in human diabetes. Diabetologia.

[41] Nozaki J ichi (2004). The endoplasmic reticulum stress response is stimulated through the continuous activation of transcription factors ATF6 and XBP1 in Ins2+/Akita pancreatic beta cells. Genes Cells.

[42] Ni M (2011). Beyond the endoplasmic reticulum: atypical GRP78 in cell viability, signalling and therapeutic targeting. Biochem J.

[43] Morita S (2017). Targeting ABL-IRE1α signaling spares ER-stressed pancreatic β cells to reverse autoimmune diabetes. Cell Metab.

[44] Herlea-Pana O (2021). Pharmacological inhibition of inositol-requiring enzyme 1α RNase activity protects pancreatic beta cell and improves diabetic condition in insulin mutation-induced diabetes. Front Endocrinol (Lausanne).

[45] Lee H (2020). Beta cell dedifferentiation induced by IRE1α deletion prevents type 1 diabetes. Cell Metab.

[46] Hodish I (2010). Misfolded proinsulin affects bystander proinsulin in neonatal diabetes. J Biol Chem.

[47] Lee YH (2003). c-Jun N-terminal kinase (JNK) mediates feedback inhibition of the insulin signaling cascade. J Biol Chem.

[48] Zhang S (2003). Fibrillogenic amylin evokes islet beta cell apoptosis through linked activation of a caspase cascade and JNK1. J Biol Chem.

[49] Abdelli S (2007). The c-Jun N-terminal kinase JNK participates in cytokine- and isolation stress-induced rat pancreatic islet apoptosis. Diabetologia.

[50] Prause M (2014). JNK1 protects against glucolipotoxicity-mediated beta cell apoptosis. PLoS One.

[51] Fukuda K (2008). c-Jun amino terminal kinase 1 deficient mice are protected from streptozotocin-induced islet injury. Biochem Biophys Res Commun.

[52] Mazzoli A (2021). JNK1 ablation improves pancreatic β cell mass and function in db/db diabetic mice without affecting insulin sensitivity and adipose tissue inflammation. FASEB Bioadv.

[53] Chan JY (2015). The balance between adaptive and apoptotic unfolded protein responses regulates β cell death under ER stress conditions through XBP1, CHOP and JNK. Mol Cell Endocrinol.

[54] Fornoni A (2008). Inhibition of c-jun N terminal kinase (JNK) improves functional beta cell mass in human islets and leads to AKT and glycogen synthase kinase-3 (GSK-3) phosphorylation. Diabetologia.

[55] Noguchi H (2007). Activation of c-Jun NH2-terminal kinase (JNK) pathway during islet transplantation and prevention of islet graft loss by intraportal injection of JNK inhibitor. Diabetologia.

[56] Noguchi H (2018). Modified cell-permeable JNK inhibitors efficiently prevents islet apoptosis and improves the outcome of islet transplantation. Sci Rep.

[57] Maedler K (2008). Glucose and leptin induce apoptosis in human beta cells and impair glucose-stimulated insulin secretion through activation of c-Jun N-terminal kinases. FASEB J.

[58] Ijaz A (2009). Inhibition of C-jun N-terminal kinase improves insulin sensitivity but worsens albuminuria in experimental diabetes. Kidney Int.

[59] Abdelli S (2009). JNK3 is abundant in insulin-secreting cells and protects against cytokine-induced apoptosis. Diabetologia.

[60] Ezanno H (2014). JNK3 is required for the cytoprotective effect of exendin 4. J Diabetes Res.

[61] Wu Y (2024). JNK3 inhibitors as promising pharmaceuticals with neuroprotective properties. Cell Adh Migr.

[62] Das M (2007). Suppression of p53-dependent senescence by the JNK signal transduction pathway. Proc Natl Acad Sci U S A.

[63] Kim WH (2005). Synergistic activation of JNK/SAPK induced by TNF-alpha and IFN-gamma: apoptosis of pancreatic beta cells via the p53 and ROS pathway. Cell Signal.

[64] Yuan H (2010). NADPH oxidase 2-derived reactive oxygen species mediate FFAs-induced dysfunction and apoptosis of β cells via JNK, p38 MAPK and p53 pathways. PLoS One.

[65] Behnke J (2015). BiP and its nucleotide exchange factors Grp170 and Sil1: mechanisms of action and biological functions. J Mol Biol.

[66] Ittner AA (2014). The nucleotide exchange factor SIL1 is required for glucose-stimulated insulin secretion from mouse pancreatic beta cells in vivo. Diabetologia.

[67] Synofzik M (2014). Absence of BiP co-chaperone DNAJC3 causes diabetes mellitus and multisystemic neurodegeneration. Am J Hum Genet.

[68] Fritz JM (2014). Deficiency of the BiP cochaperone ERdj4 causes constitutive endoplasmic reticulum stress and metabolic defects. Mol Biol Cell.

[69] Ladiges WC (2005). Pancreatic beta cell failure and diabetes in mice with a deletion mutation of the endoplasmic reticulum molecular chaperone gene P58IPK. Diabetes.

[70] Schmitz A (1995). In vivo iodination of a misfolded proinsulin reveals co-localized signals for Bip binding and for degradation in the ER. EMBO J.

[71] Gorasia DG (2016). A prominent role of PDIA6 in processing of misfolded proinsulin. Biochim Biophys Acta.

[72] Zhang L (2009). GRP78, but not protein-disulfide isomerase, partially reverses hyperglycemia-induced inhibition of insulin synthesis and secretion in pancreatic {beta} cells. J Biol Chem.

[73] Teodoro-Morrison T (2013). GRP78 overproduction in pancreatic beta cells protects against high-fat-diet-induced diabetes in mice. Diabetologia.

[74] Cunha DA (2009). Glucagon-like peptide-1 agonists protect pancreatic beta cells from lipotoxic endoplasmic reticulum stress through upregulation of BiP and JunB. Diabetes.

[75] Ye R (2010). Grp78 heterozygosity promotes adaptive unfolded protein response and attenuates diet-induced obesity and insulin resistance. Diabetes.

[76] Shen J (2017). GRP78 haploinsufficiency suppresses acinar-to-ductal metaplasia, signaling, and mutant Kras-driven pancreatic tumorigenesis in mice. Proc Natl Acad Sci U S A.

[77] Stamateris RE (2016). Glucose induces mouse β cell proliferation via IRS2, MTOR, and Cyclin D2 but not the insulin receptor. Diabetes.

[78] Dobin A (2013). STAR: ultrafast universal RNA-seq aligner. Bioinformatics.

[79] Kucukural A (2019). DEBrowser: interactive differential expression analysis and visualization tool for count data. BMC Genomics.

[80] Carpenter AE (2006). CellProfiler: image analysis software for identifying and quantifying cell phenotypes. Genome Biol.

