# Supplementary material for: JNK and p53 cause human and mouse β cell death during excessive unfolded protein response
Source: J Clin Invest. 2026 Aug 3;136(15):e193035. doi: 10.1172/JCI193035 (PMC13430013; doi:10.1172/JCI193035)
Supplement: Supplemental data [file jci-136-193035-s023.pdf]

Name of the Reagent	Company	Catalogue Number
10x HBSS	Gibco (Thermo Scientific)	14065-056
Sodium Bicarbonate	Sigma-Aldrich	S6014-500G
New Born Calf Serum	Sigma- Aldrich	N4637-500mL
Collagenase P	Roche	11249002001
RPMI 1640 Medium, No glucose	Gibco (Thermo Scientific)	11879020
1x DPBS	Gibco (Thermo Scientific)	14190-144
Trypsin EDTA (0.05%)	Gibco (Thermo Scientific)	25300-054
Fetal Bovine Serum	Sigma	F0926-500mL
USDA Import Tested Fetal Bovine Serum	Cytiva	SH30910.03
D (+) Glucose	Sigma	G7021-1KG
Beta-2-mercaptoethanol	Sigma	M3148-25mL
Penicillin Streptomycin Solution, 100X	Corning	30-002-Cl
Dimethyl Sulfoxide	Sigma	D8418-100ML
Paraformaldehyde 16% solution, EM Grade	Electron microscopy Sciences	15710-S
Formalin Solution	Sigma- Aldrich	HT501128-4L
Xylene, Histological Grade	Sigma- Aldrich	534056-4L
Ethanol 200 Proof	Decon Labs	2701
Goat Serum	Gibco (Thermo Scientific)	16210072
10X PBS	Fisher	BP3994
Triton X-100 for Molecular biology	Sigma- Aldrich	T8787-100mL
Bovine Serum Albumin	Equitech-Bio	BAH66
Hydrochloric Acid	Fisher	A144-500
3,3'-Diaminobenzidine (DAB) enhanced liquid substrate system tetrahydro-cholride	Sigma	D3939-1Set
RNA/DNA/Protein Purification plus kit	Norgen Biotek Corp	47700
Ethyl-alcohol, pure	Sigma- Aldrich	7023-500mL
SuperScript IV Vilo Master Mix	Thermo Scientific	11756050
PerfeCTa Syber Green FastMix, ROX	Quanta bio	95073-05K
p-APMSF, Hydrochloride	Sigma	178281-5MG
Dithiothreitol	Sigma	10197777001
EASYpack Protease Inhibitor Cocktail	Roche	5892970001
PHOSSTOP, PHOSPHATASE INHIBITOR	Roche	4906845001
Mini-Protean TGX gels	Bio-Rad	4561096
Methanol	Fisher	A412-4
10% SDS Solution	Bio-Rad	1610416
10X Tris/Glycine Buffer	Bio-Rad	1610771
Lane Marker Reducing Sample	Thermo Scientific	39000
Precision Plus, protein dual color standards	Bio-Rad	1610374
SuperSignal West Femto Maximum Sensitivity Substrate	Thermo Scientific	34096

Pierce ECL western	Thermo Scientific	32106
ECL Prime Western Blotting Detection	Cytiva	RPN2236
Nuclease free Water	Sigma	W4502-1L
QuickExtract DNA Extraction Solution	Lucigen Corp.	QE09050
Ultrapure Agarose	Invitrogen	16500-50
Ethidium Bromide	Teknova	E3050
Sybr safe DNA gel Stain	Invitrogen	S33102
Kapa2G Fast HS Genotyping Mix	Kapa biosystems	KK5621
GeneRuler 100bP Plus DNA Ladder	Life technologies	SM0324
10X TAE Buffer	Corning	46-010-CM

Supplementary Table 1: General reagents and sources

Name	Vendor	Cat No	MOI
Ad-CMV-iCre	Vector Biolabs	1045	20
Ad-CMV-LacZ	Vector Biolabs	1080	10/20
Ad-GFP-U6-ShRNA	Vector Biolabs	1122	20
Ad-m-HSPA-ShRNA	Vector Biolabs	AShADV-261744	20
Ad-h-HSPA-ShRMA	Vector Biolabs	ShADV-211643	20
Ad-h-HSPA5	Vector Biolabs	ADV-211642	10

Supplementary Table 2: Adenoviruses

Reagent	Company	Cat #	Activity
JNK-IN-8	EMD Millipore	420150	Ex vivo pan JNK inhibitor
APY29	Med Chem Express	HY-17537	IRE1 Kinase Inhibitor
STF-083010	Med Chem Express	HY-15845	IRE1 Nuclease Inhibitor
SB202190	Selleck Chem	S1077	Pan p38 MAPK Inhibitor
Pifithrin-α (PFTα) HBr	Selleck Chem	S2929	Pan p-53 Inhibitor
D-JNKI-1	Med Chem Express	HY-P0069	In vivo JNK Inhibitor
Atf6 Inhibitor, AEBSF	Thermo Fisher	78431	Serine protease inhibitor
GSK2606414	Med Chem Express	HY-18072	PERK Inhibitor
4μ8c	Selleck Chem	S7272	Pan IRE1 Inhibitor

Supplementary Table 3: Chemical inhibitors for in vitro and in vivo studies

Isolation ID	Diabetic	Gender	Age	BMI	Ethnicity	Cause of Death
RRID: SAMN08768702	No	F	59	22	White	Cerebrovascular/ stroke
RRID: SAMN08768738	No	M	59	26.8	White	Anoxia
RRID: SAMN08768735	No	M	68	29.7	White	Cerebrovascular/ stroke
RRID: SAMN08768731	No	M	60	31.3	White	Head trauma
RRID: SAMN08768751	No	M	60	37.9	White	Anoxia
RRID: SAMN18200483	No	F	48	47.6	Black/African American	Cerebrovascular/ stroke
RRID: SAMN22818629	No	F	45	21.7	White	Anoxia
RRID: SAMN22997021	No	M	44	42	White	Head Trauma-
RRID: SAMN23079315	No	F	37	30.2	White	Anoxia
RRID: SAMN23958504	No	M	42	37.9	White	Cerebrovascular/ stroke
RRID: SAMN24579752	No	M	55	24.9	Black/African American	Cerebrovascular/ stroke
RRID: SAMN26646319	No	M	20	20.5	Black/African American	Head Trauma
RRID: SAMN27022590	No	F	44	30.7	White	Cerebrovascular/ stroke

Supplementary Table 4: Donor information for human islets used for ex vivo studies

Slide Number	Isolation ID	Diabetic	Gender	Age	BMI	Ethnicity	Cause of Death
982952	RRID: SAMN22982952	No	M	61	29.3	Hispanic /Latino	Cerebrovascular/stroke
84832	RRID: SAMN22784832	Yes	M	60	41.3	Hispanic /Latino	Cerebrovascular/stroke
84816	RRID: SAMN22784816	Yes	F	62	38.5	Black /Afr. Am.	Cerebrovascular/stroke
84814	RRID: SAMN22784814	No	F	59	28.3	Hispanic /Latino	Cerebrovascular/stroke
84651	RRID: SAMN22784651	Yes	M	53	26	White	Anoxia
84623	RRID: SAMN22784623	No	M	61	33.3	Hispanic /Latino	Cerebrovascular/stroke
84617	RRID: SAMN22784617	Yes	M	63	31.7	White	Cerebrovascular/stroke
83447	RRID: SAMN22783447	Yes	F	56	23.1	White	Anoxia
81951	RRID: SAMN22781951	No	F	56	26.6	White	Cerebrovascular/stroke
81928	RRID: SAMN22781928	No	M	58	46.8	Black /Afr. Am.	Cerebrovascular/stroke

Supplementary Table 5: Donor information for human pancreas sections

Antibody	Catalog	Company	Dilution for WB	Dilution for IF/IHC
Rb anti-glucagon	2760	Cell signaling		1:250
rat anti-somatostatin	Ab30788	Abcam		1:100
Rb anti-pJNK	4668	Cell signaling	1:1000	1:200
Rb anti-p-p53	9284	Cell signaling	1:1000	1:200
TUNEL	G3250	Promega	**-**	**-**
Rb anti-p-IRE1	ab124945	Abcam	1:1000	**-**
Rb anti-c-Caspase 3	9661	Cell signaling	1:500	**-**
mouse Chop	2895	Cell signaling	1:500	**-**
mouse anti-Grp78	610978	BD Biosciences	1:5000	1:500
Anti-Actin	MAB1501R	Millipore Sigma	1:5000	**-**
GP anti-insulin	LS-C346763-10	LS bio	**-**	1:500

Supplementary Table 6: Antibodies for western blots and immunostaining

Primer	Forward (5’-3’)	Reverse (5’-3’)
Grp78	TCTCCACGGCTTCCGATAAT	GTACCTTTGTCTTCAGCTGTCACTC
Grp94	TGATGAAGTCGACGTGGATG	TCCTGTTCACTTCAGCTTGG
CalR	CTGCATAGGCCTCATCATTG	AGTTTTGCTGTACTGGGCCT
Chop	CAC ATC CCA AAG CCC TCG	CTC AGT CCC CTC CTC AGC
Atf3	GCTGCCAAGTGTCGAAACAAG	CAGTTTTCCAATGGCTTCAGG
Bak1	GTGACCTGCTTTTTGGCTGAT	GGTCTCTACGCAAATTCAGGG
Trib3	GGCCTTATATCCTTTTGGAACGA	CGCTGGCAGGGTACACCTT
Phlda3	CCGTGGAGTGCGTAGAGAG	TCGGTCACTAGCGTGAAGTAG
Sdc1	AGGATGGAACTGCCAATCAG	ATCCGGTACAGCATGAAAGC
Ier5	GGACGACACCGACGAGGAG	GCTTTTCCGTAGGAGTCCCG
Nfatc2	TCATCCAACAACAGACTGCCC	GGGAGGGAGGTCCTGAAAACT
Pvr	GGGTGGGGATATACGTGTGC	GTTCCTCAGATCCTGTTGGGC
Txnip	CGAGTCAAAGCCGTCAGGAT	TTCATAGCGCAAGTAGTCCAAGGT
Map1lc3b	TTATAGAGCGATACAAGGGGGAG	CGCCGTCTGATTATCTTGATGAG
Lgals3	AGACAGCTTTTCGCTTAACGA	GGGTAGGCACTAGGAGGAGC
Wee1	GTCGCCCGTCAAATCACCTT	GAGCCGGAATCAATAACTCGC
Fosb	TTTTCCCGGAGACTACGACTC	GTGATTGCGGTGACCGTTG
Hyou1	TAGCCAGGTGTTCTCGAAGC	GACTAAGGAGGCTGGGATGC
HerpUD1	CAACAGCAGCTTCCCAGAAT	CCGCAGTTGGAGTGTGAGT
Pdia4	ATCGCCAAGATGGATGCTAC	CTTGGTCCTGCTCCTCTTTG
Sel1L	GTCGTCTTTTGGCAGCATCT	GATCTCCGAATCCAAGCAG
Ssr3	ACCAGAACCAGGAACAGAGTG	CCGGAAGGAGAAAGACGAA
Sec24D	GGAGAGGTCTTTGTTCCTTTGTT	GTCTCTGTTCTTGAGCTTCCC
Erdj4	TGAATTTGCAGAGGTTTCACTG	CAAACTCAGCCCGACACATA
Atf4	GGACAGATTGGATGTTGGAGAAAATG	GGAGATGGCCAATTGGGTTCAC
Asna	CGCTGGAACCCACGCTTAG	CAGGTGGTCTTACCAACGCC
Asns	TGACCCGCTGTTTGGAATG	CTGTAGCGCCTTGTGGTTGTA
iCre	TGTCTGGTGTGGCTGATGAC	TTGGCACCATAGATCAGGCG

Supplementary Table 7: Mouse real-time PCR primers

Primer	Forward (5'-3')	Reverse (5'-3')
GRP78	CATCAAGTTCTTGCCGTTCA	ATGTCTTTGTTTGCCCACCT
GRP94	GCTGACGATGAAGTTGATGTGG	CATCCGTCCTTGATCCTTCTCTA
CHOP	AGAACCAGGAAACGGAAACAGA	TCTCCTTCATGCGCTGCTTT
ATF3	CACCTTTGCCATCGGATGTCC	CTTTCCCGCCGCCTCCTT
BAK1	TCATCGGGGACGACATCAAC	CAAACAGGCTGGTGGCAATC
PHLDA3	CCGTGGAGTGCGTGGAGAC	CTAGGGTGATCTGGGCGTTCC
SDC1	GCTCTGGGGATGACTCTGAC	GTATTCTCCCCCGAGGTTTC
IER5	AGGCTCATCGCATCGTCAG	CGCTCAGGTAGACTTGGCG
NFATC2	GAGCCGAATGCACATAAGGTC	CCAGAGAGACTAGCAAGGGG
PVR	TGGAGGTGACGCATGTGTC	GTTTGGACTCCGAATAGCTGG
TXNIP	ACAGAAAAGGATTCTGTGAAGGTGAT	GCCATTGGCAAGGTAAGTGTG
MAP1LC3B	GATGTCCGACTTATTCGAGAGC	TTGAGCTGTAAGCGCCTTCTA
LGALS3	ATGGCAGACAATTTTTCGCTCC	GCCTGTCCAGGATAAGCCC
WEE1	AGGGAATTTGATGTGCGACAG	CTTCAAGCTCATAATCACTGGCT
FOSB	GCTGCAAGATCCCCTACGAAG	ACGAAGAAGTGTACGAAGGGTT
HYOU1	AAGGCTCACTTCAACCTGGA	TCTCCTCTTCCTCCTGGACA
HERPUD1	GAGCCTGCTGGTTCTAATCG	GAAAGCTGAAGCCACCCATA
PDIA4	CTCCACCAGAAGTCACGCTT	GGGGCAAGTTTCTTGCAGTG
SSR3	GGATTTCAGCCGCAATCTCTC	TGCCATATTCGCCAGTATAACC
SEC24D	CCGCCACTAGGGGAATGTTG	AGGGTTGGTATGGTGCATGTG
ERDJ4	TCTTAGGTGTGCCAAAATCGG	TGTCAGGGTGGTACTTCATGG
ATF4	CTCCGGGACAGATTGGATGTT	GGCTGCTTATTAGTCTCCTGGAC
ASNA	GCCTACCAAGGTCAAAGGCTA	CGTGTCAAATACCACCACCG
ASNS	GGAAGACAGCCCCGATTTACT	AGCACGAACTGTTGTAATGTCA

Supplementary Table 8: Human real-time PCR primers

Ins1Cre+ mTmG+

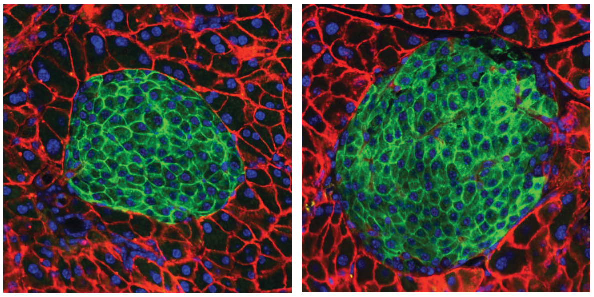

Supplementary Figure 1. Efficient recombination of the *Ins1-cre* strain in the Alonso colony. To assess recombination efficiency, *Gt(ROSA)26Sor tm4(ACTB-tdTomato,-EGFP)Luo /J* ("mTmG") recombination reporter mice were imported from JAX and mated to *Ins1cre* mice in the Alonso colony. Paraffin sections of pancreas from *Ins1cre+mTmG+* mice showed most islet cells with evidence of recombination (green color; mG) while exocrine pancreas and some islet cells, presumably non-beta cells, remained non-recombined (red color; mT).

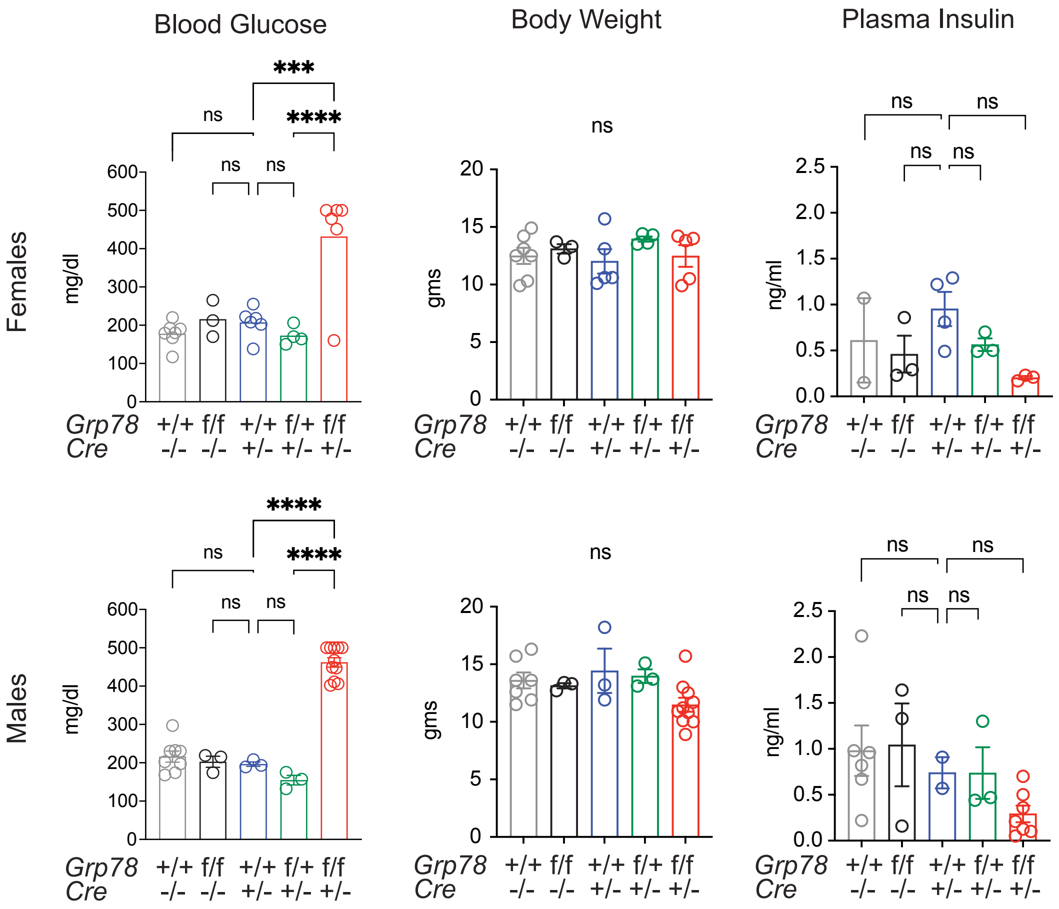

Supplementary Figure 2. Male and female *Grp78^f/f^Ins1cre^+/-^* pups had similar blood glucose, body weight, and plasma insulin. Data are shown for pups at 4 weeks of age. Statistics by one-way ANOVA. ns, not significant; ***p<0.001; ****p<0.0001.

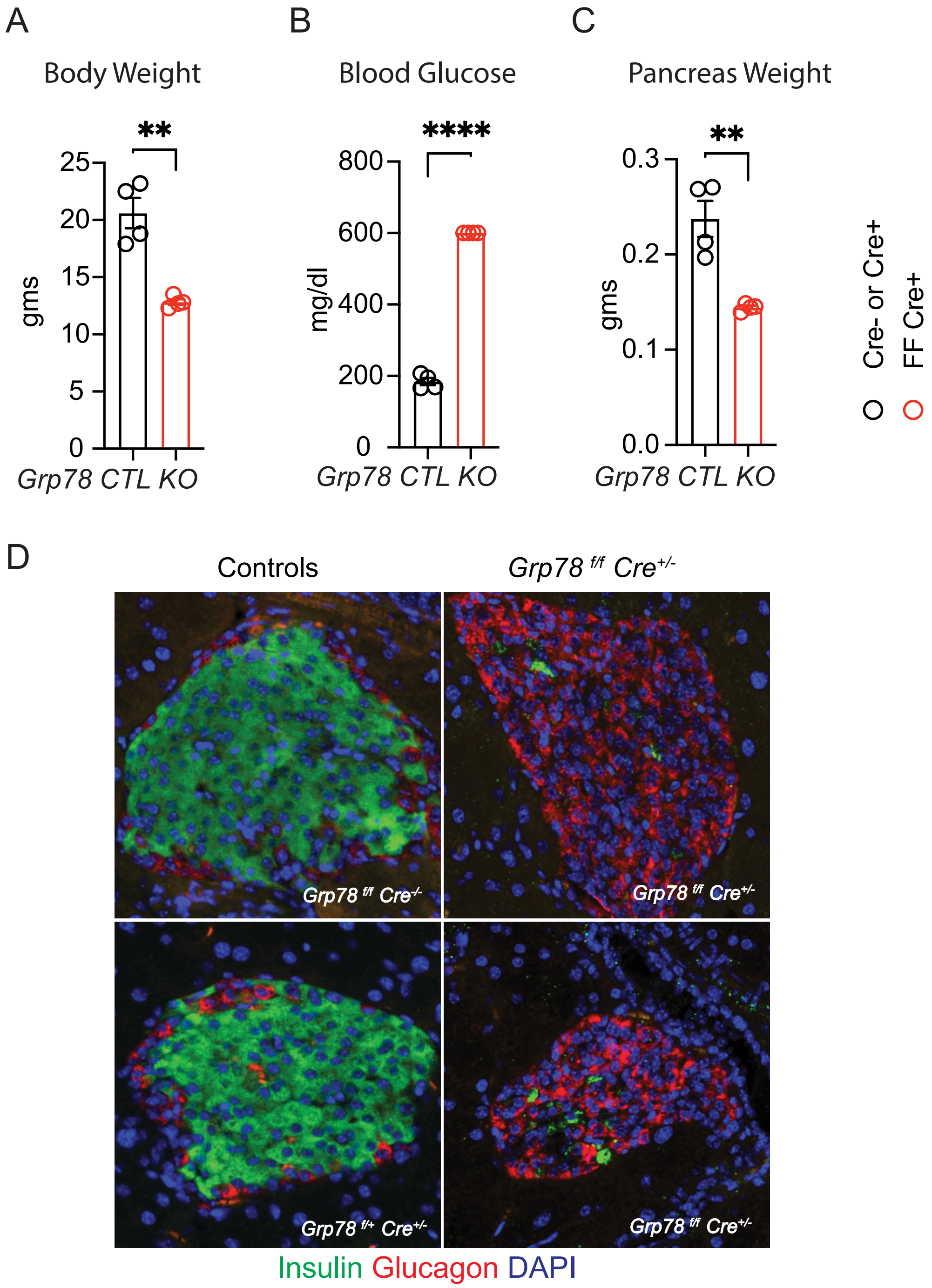

Supplementary Figure 3. Metabolic phenotype of lifelong beta cell *Grp78* deletion, tested at 8 weeks of age. *Grp78f/f-Ins1cre*+ mice and controls were kept until 8 weeks of age and then assessed for body weight (A), random blood glucose (B), and pancreas weight (C). Histological analysis of paraffin sections from 8-week old mice immunostained for insulin (green), glucagon (red) and dapi (blue) showed a marked reduction in beta cells per islet in *Grp78* deletion mice (D). Statistics by Student's T-test: **p<0.01; ****p<0.0001.

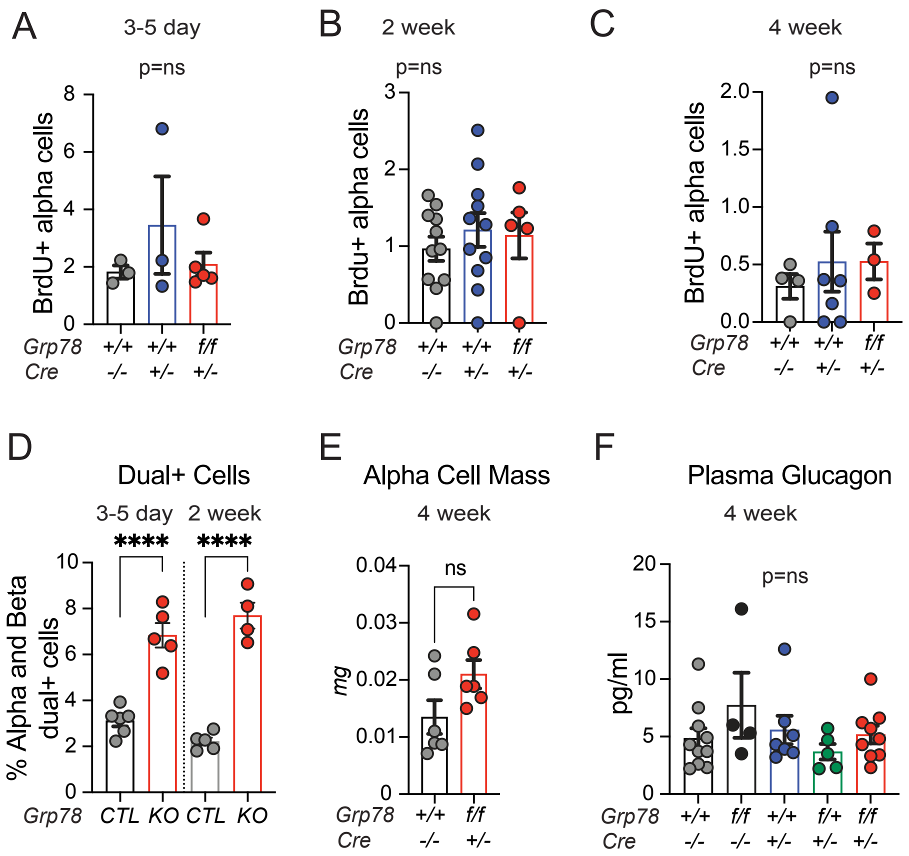

Supplementary Figure 4. Grp78 deletion did not increase alpha cell proliferation, alpha cell mass, or circulating glucagon levels. Sections from *Grp78* deleted mice and controls were immunostained for insulin, glucagon, BrdU, and dapi, and the percentage of glucagon+ cells that were BrdU+ was quantified (A-C). From the same sections, the percentage of insulin+ cells that were also glucagon+ was quantified (D). Using whole-pancreas scanned images of sections stained for glucagon and dapi, the fractional area of the total pancreatic area that was glucagon+ was estimated using Strataquest software, and the % glucagon area was multiplied by the wet weight of the pancreas to estimate alpha cell mass (E). Circulating glucagon levels were measured by ELISA (F). Statistics by one-way ANOVA or Student's T-test (E). ns, not significant; ****p<0.0001.

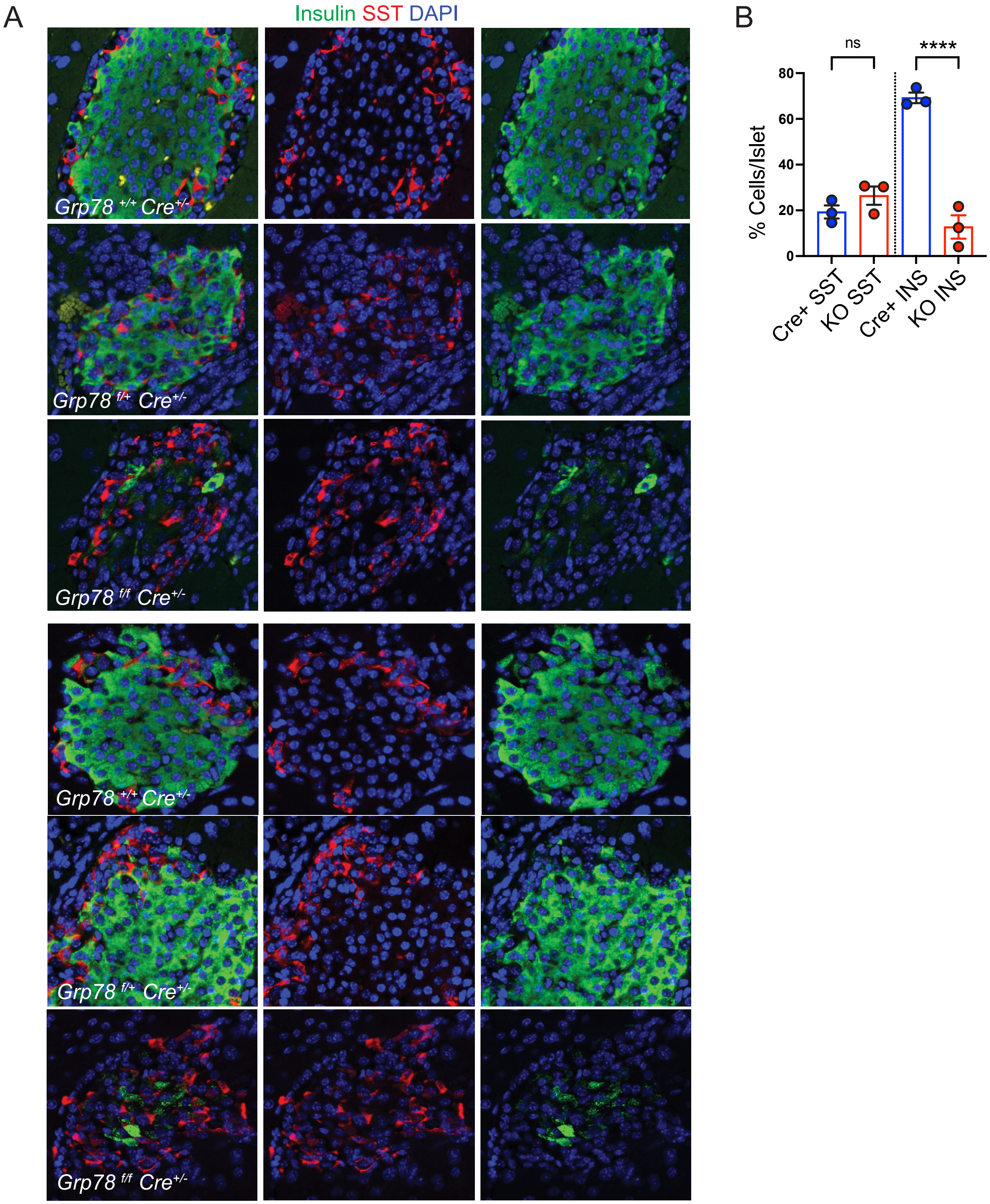

Supplementary Figure 5. Quantification of somatostatin+ cells in pancreas sections from 4-week-old mice. Paraffin sections from 4-week-old mice of the indicated genotypes were labeled for insulin (green), somatostatin (red), and dapi (blue). (A) Top and bottom panels show representative images of islets from each genotype; the left column shows the merge, middle column shows somatostatin/dapi only, and the right column shows insulin/dapi only. (B) Quantification of % islet cells of each type confirms the reduction in insulin+ cells at 4 weeks; the % islet cells that were SST+ was not altered in KO islets. Statistics by one-way ANOVA. ns, not significant; ****p<0.0001.

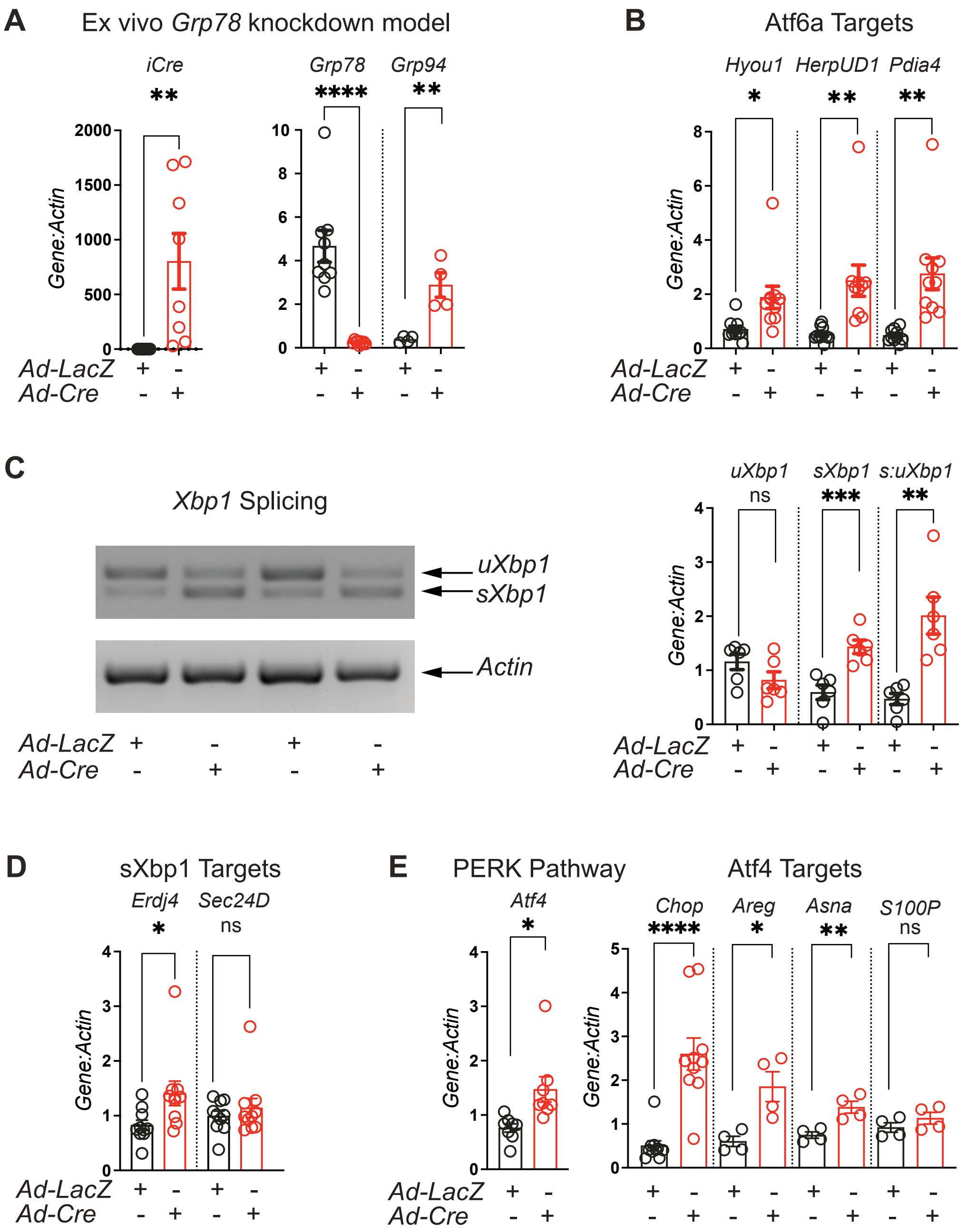

Supplementary Figure 6. Ex vivo GRP78 depletion activated all three canonical UPR pathways. *Grp78-f/f* islets from male and female mice were dispersed, transduced with *Ad-LacZ* or *Ad-iCre*, cultured for 72 hours in 15mM glucose for all panels. RNA was isolated, cDNA synthesized, and pQCR performed for the indicated genes. **A**: Ex vivo *Grp78* knockdown is effective, with increased *iCre* expression, decreased *Grp78* expression, and compensatory increase of *Grp94*. **B-E**: *Grp78* knock-down increased ATF6 targets (**B**), *Xbp1* splicing (**C**), and PERK pathway related gene expression (**D-E**). Statistics by one-way ANOVA. ns, not significant; ***p<0.001; ****p<0.0001.

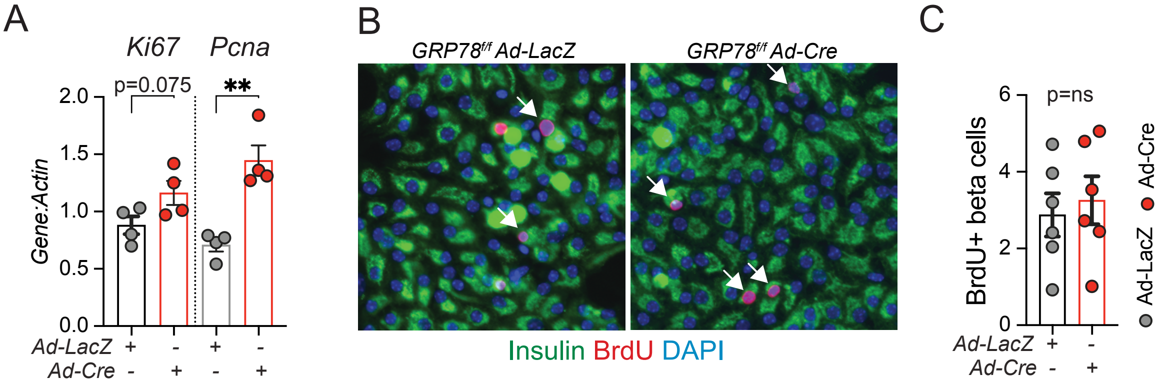

Supplementary Figure 7. Ex vivo GRP78 depletion increases *Pcna* transcript abundance but does not increase S-phase entry. *Grp78-f/f* islets were dispersed, transduced with *Ad-LacZ* or *Ad-iCre*, and cultured for 72 hours in 15mM glucose. Cultures were either harvested for qPCR (A) or labeled for BrdU, Insulin, and Dapi, imaged, and counted (B-C). Statistics by Student's T-test. ns, not significant; **p<0.01.

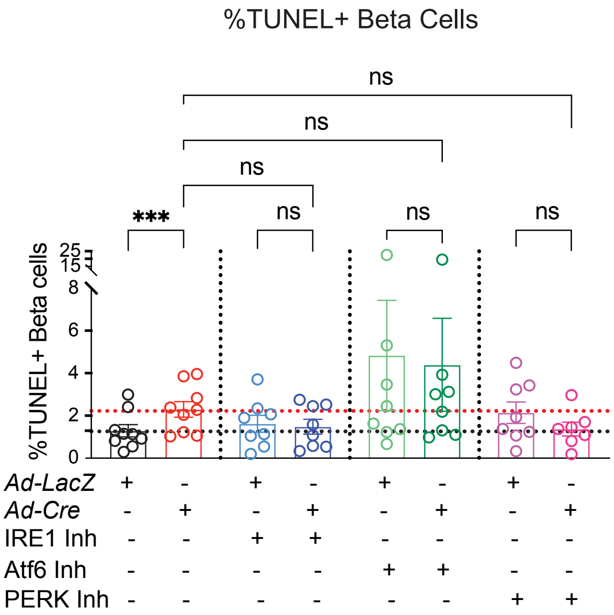

Supplementary Figure 8. Impact of UPR pathway inhibitors on mouse beta cell apoptosis during ex vivo GRP78 depletion, including the conditions in which inhibitors were added to *Ad-LacZ* (not knocked down) cultures. *Grp78-f/f* islets from male and female mice were dispersed, transduced with *Ad-LacZ* or *Ad-iCre*, cultured for 72 hours in 15mM glucose in the presence or absence of the indicated pathway inhibitors. Cultures were labeled for TUNEL, Insulin, and Dapi as in Figure 5, imaged, and counted. Statistics by one-way ANOVA. ns, not significant; ***p<0.001.

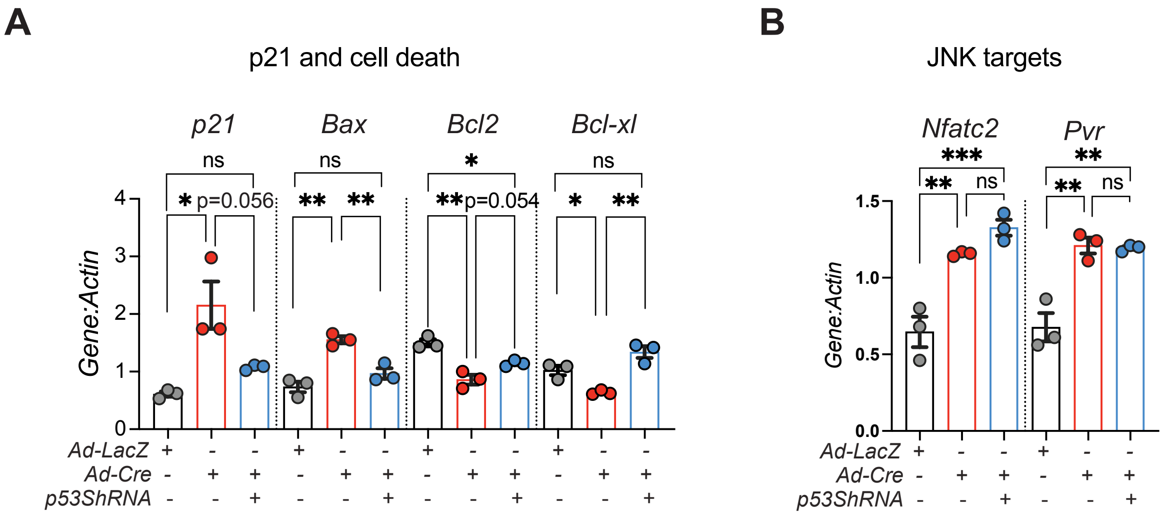

Supplementary Figure 9. Genetic knockown of p53 using shRNA reversed cell death associated changes in *Bax*, *Bcl2*, *Bcl-xl*, as well as *p21*, without changing JNK target expression, during ex vivo GRP78 depletion. *Grp78-f/f* islets were dispersed, transduced with *Ad-LacZ* or *Ad-Cre* with or without *Ad-sh-p53*, cultured for 72 hours in 15mM glucose, then harvested for qPCR. Statistics by one-way ANOVA. ns, not significant; *p<0.05; **p<0.01; ***p<0.001.

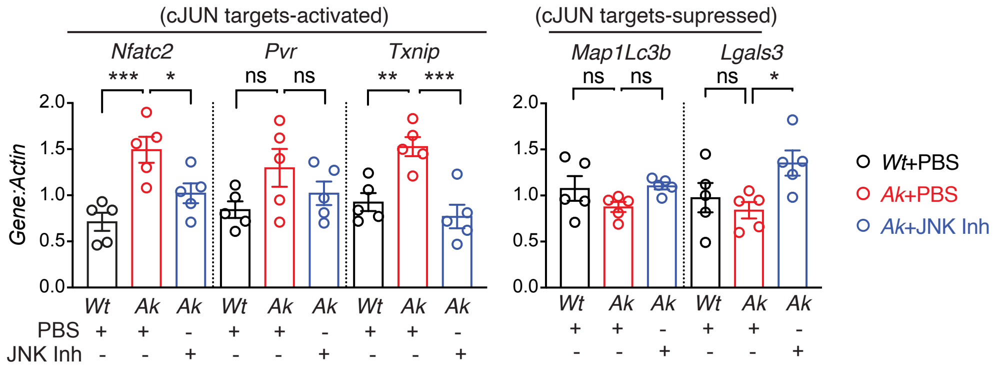

Supplementary Figure 10. Evidence for systemic activity of JNK inhibitor after 5 days of injections in *Akita* mice. Liver was isolated after the experimental paradigm shown in Figure 8A, then RNA was isolated and qPCR performed. (A) cJUN targets known to be induced by c-JUN were increased in *Akita* mice injected with PBS control, but similar to control animals in *Akita* mice injected with JNK inhibitor. (B) cJUN targets known to be suppressed by cJun were slightly down in the livers of *Akita* mice injected with PBS, and similar to controls in *Akita* mice injected with JNK inhibitro. Statistics by one-way ANOVA. ns, not significant; *p<0.05; **p<0.01; ***p<0.001.

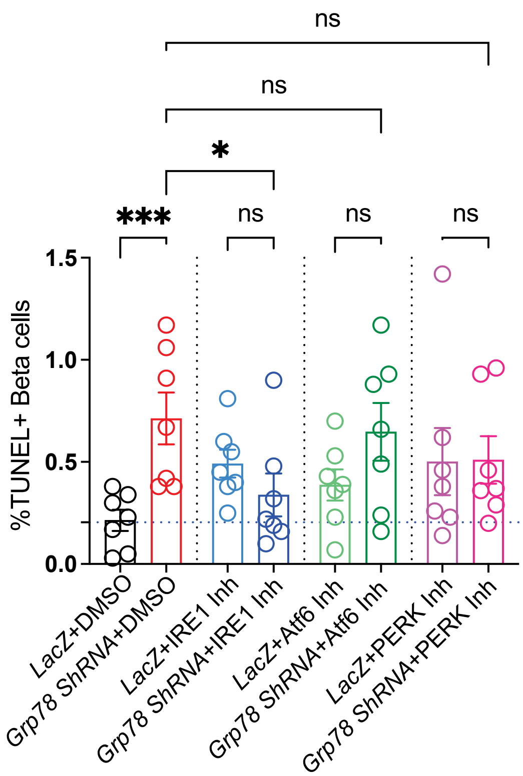

Supplementary Figure 11. Impact of UPR pathway inhibitors on beta cell apoptosis in human beta cells during ex vivo GRP78 depletion. Human islets were dispersed, transduced with *Ad-LacZ* or *Ad-sh-GRP78*, cultured for 72 hours in 15mM glucose in the presence or absence of the indicated pathway inhibitors. Cultures were labeled for TUNEL, Insulin, and Dapi, imaged, and counted as in Figure 5A-B. Data are presented as raw counts. Statistics by one-way ANOVA. ns, not significant; *p<0.05; ***p<0.001.
