# Supplementary material for: JNK and p53 cause human and mouse β cell death during excessive unfolded protein response
Source: J Clin Invest. 2026 Aug 3;136(15):e193035. doi: 10.1172/JCI193035 (PMC13430013; doi:10.1172/JCI193035)

Fig 4a

Grp78

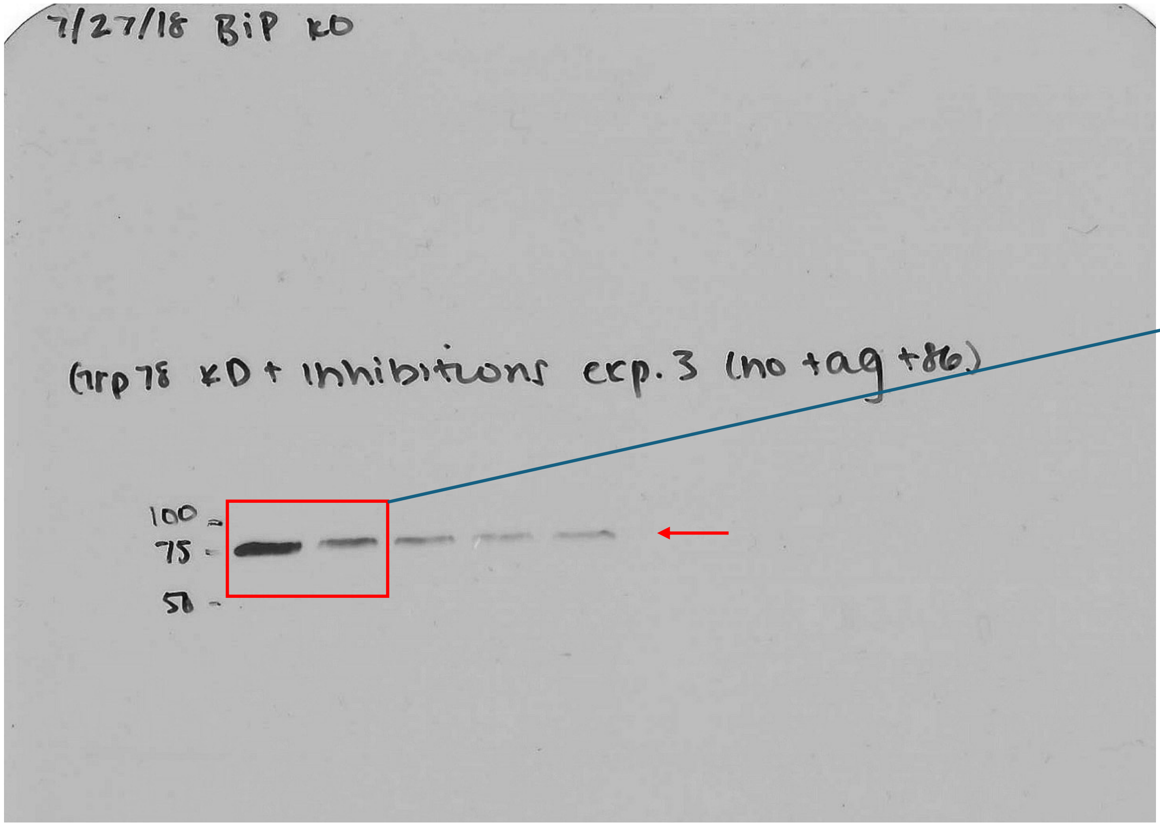

GRP78

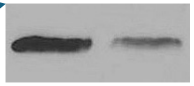

Lane 1 MWM
Lane 2 Grp78$^{f/f}$ Ad-LacZ (DMSO)
Lane 3 Grp78$^{f/f}$ Ad-Cre (DMSO)
Lane 4 Grp78$^{f/f}$ Ad-Cre+Atf6 Inh (AEBSF)
Lane 5 Grp78$^{f/f}$ Ad-Cre+IRE1 Inh (4u8c)
Lane 6 Grp78$^{f/f}$ Ad-Cre+PERK Inh (GSK2606414)

Fig 4a

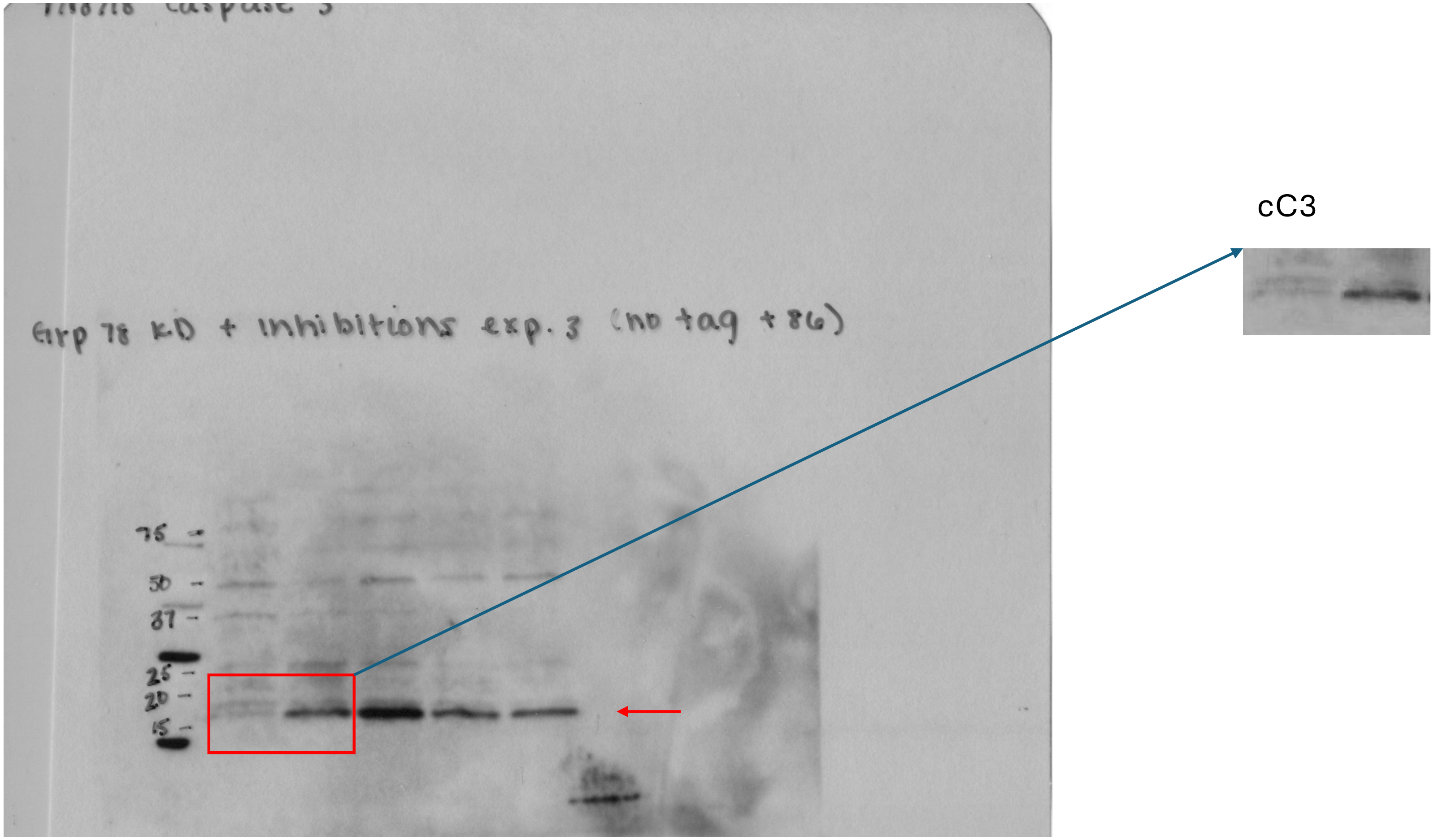

Fig 4a

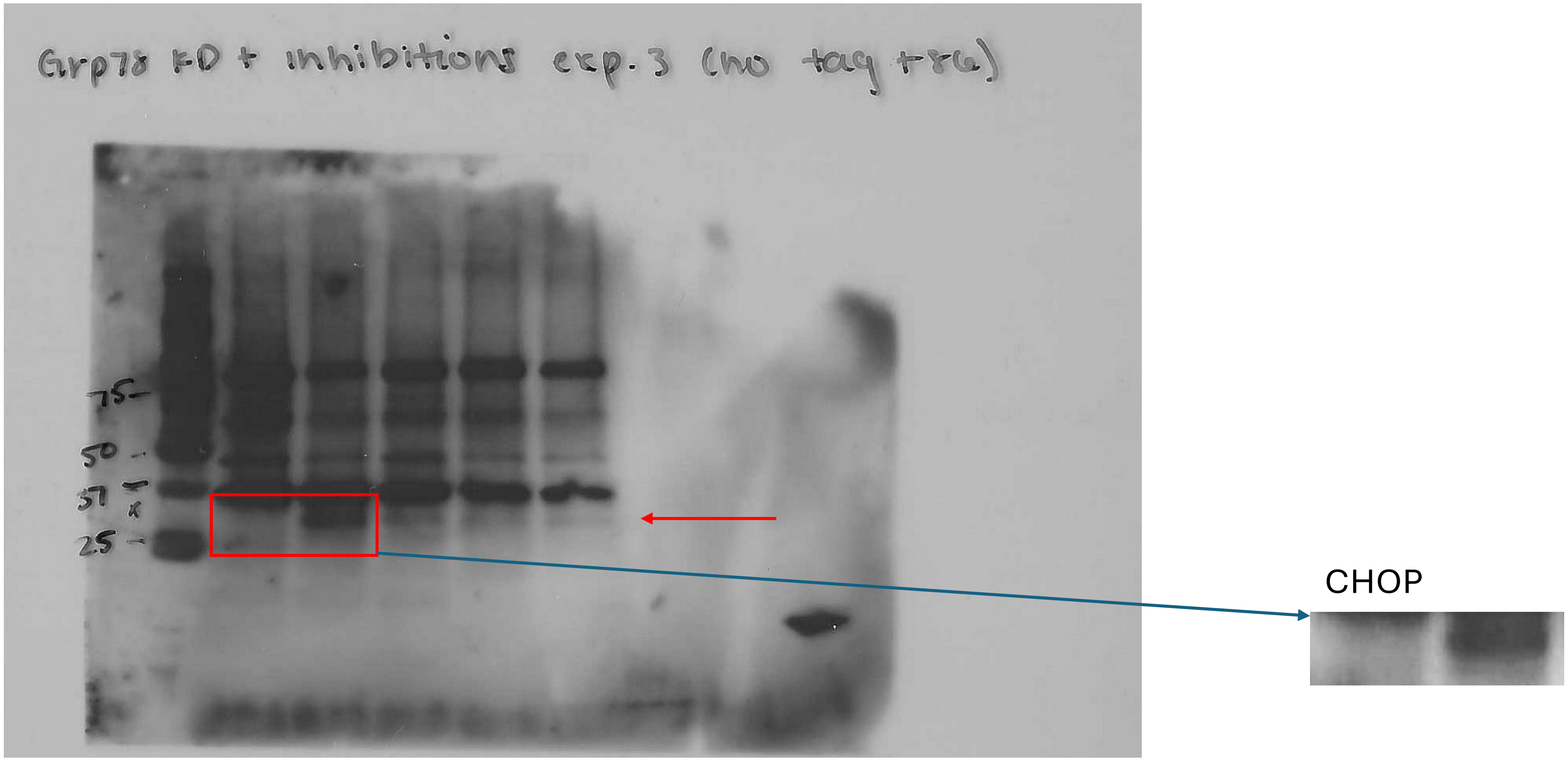

Fig 4a

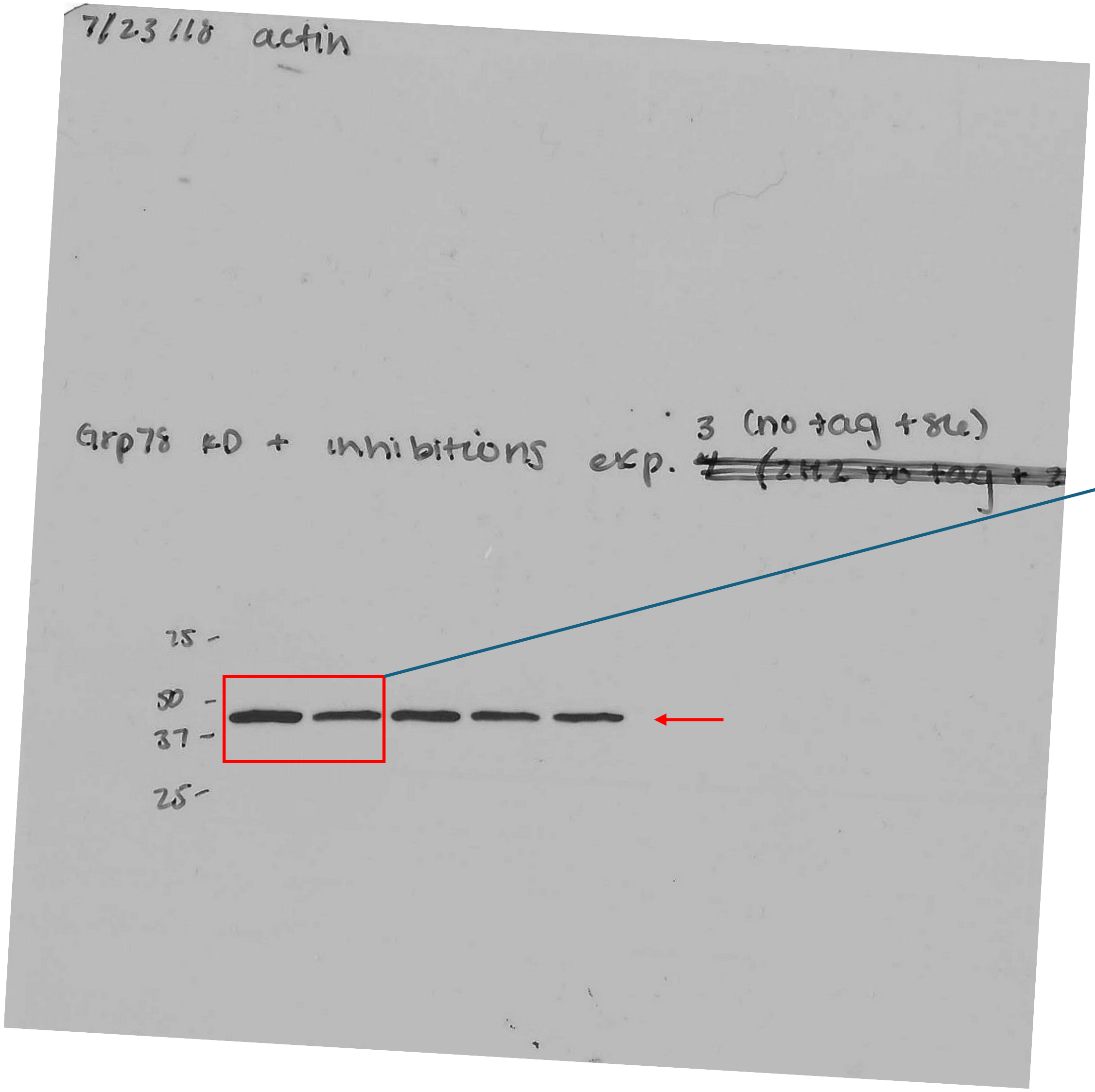

ACTIN

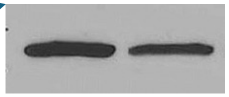

Fig 5c

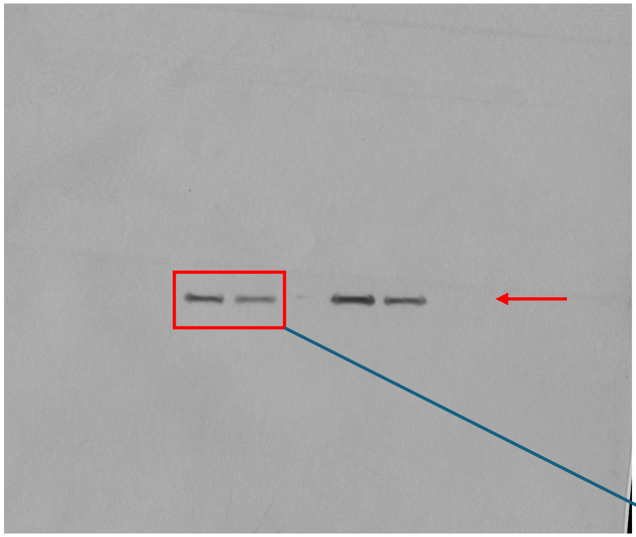

Lane 1-MWM
Lane 2-Grp78$^{f/f}$ Ad-LacZ
Lane 3-Grp78$^{f/f}$ Ad-iCre
Lane 4-MWM
Lane 5-Grp78$^{f/f}$ Ad-LacZ
Lane 6-Grp78$^{f/f}$ Ad-iCre

Labelled-higher exposure

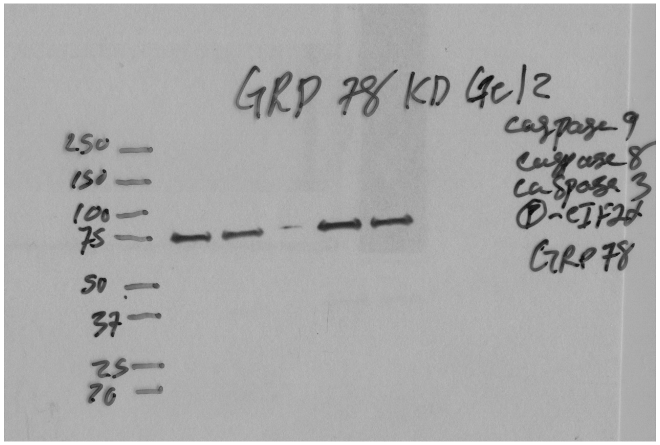

GRP78

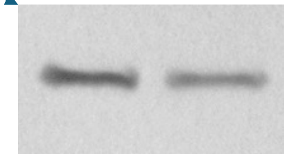

Fig 5c

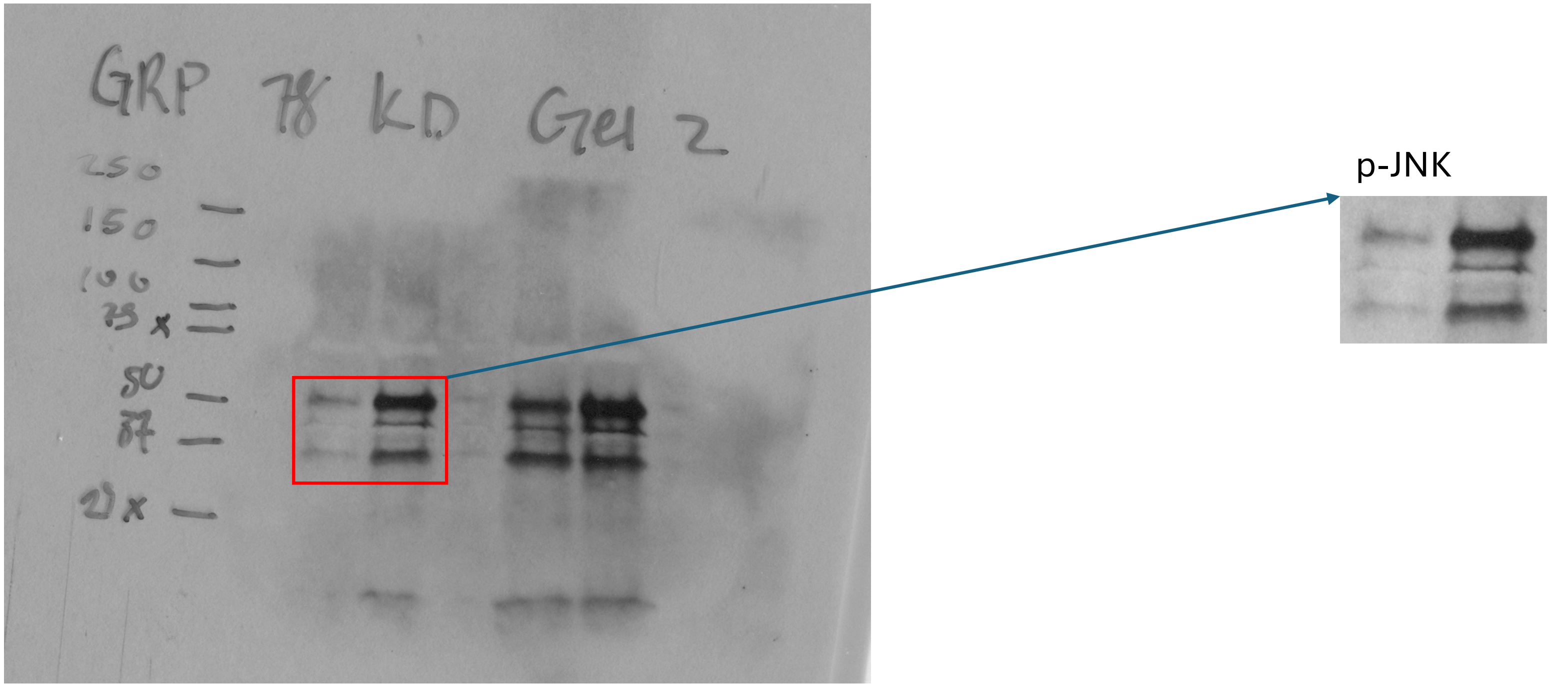

Fig 5c

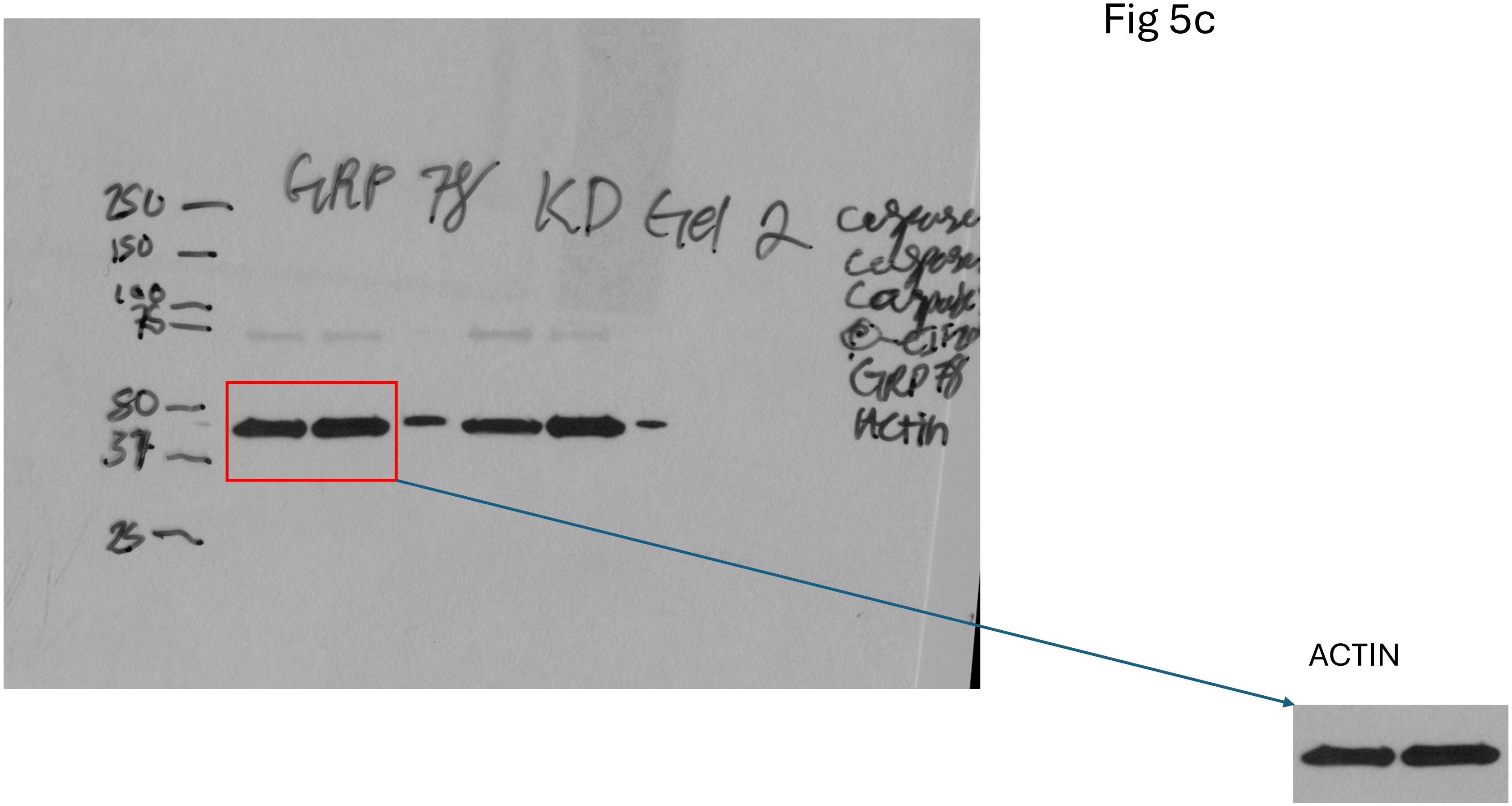

Fig 5e

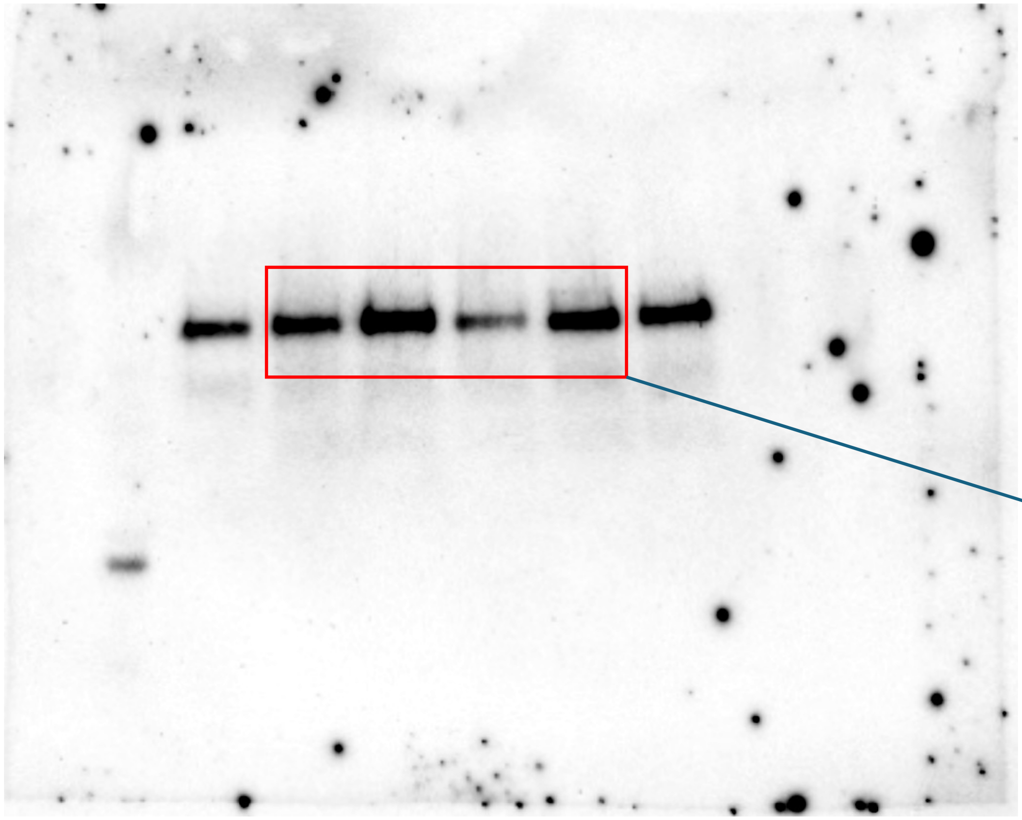

Lane 1-MWM
Lane 2-5mM Glu Grp78$^{f/f}$ Ad-LacZ+DMSO
Lane 3-15mM Glu Grp78$^{f/f}$ Ad-LacZ+DMSO
Lane 4-15mM Glu Grp78$^{f/f}$ Ad-iCre+DMSO
Lane 5-15mM Glu Grp78$^{f/f}$ Ad-iCre+APY 29 (IRE1 Kinase Inh)
Lane 6-15mM Glu Grp78$^{f/f}$ Ad-iCre+STF-083010 (IRE1 Nuclease Inh)
Lane 7-15mM Glu Grp78$^{f/f}$ Ad-iCre+JNK Inh

pIRE1

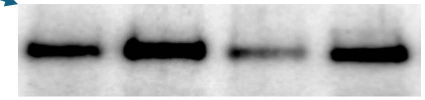

Fig 5e

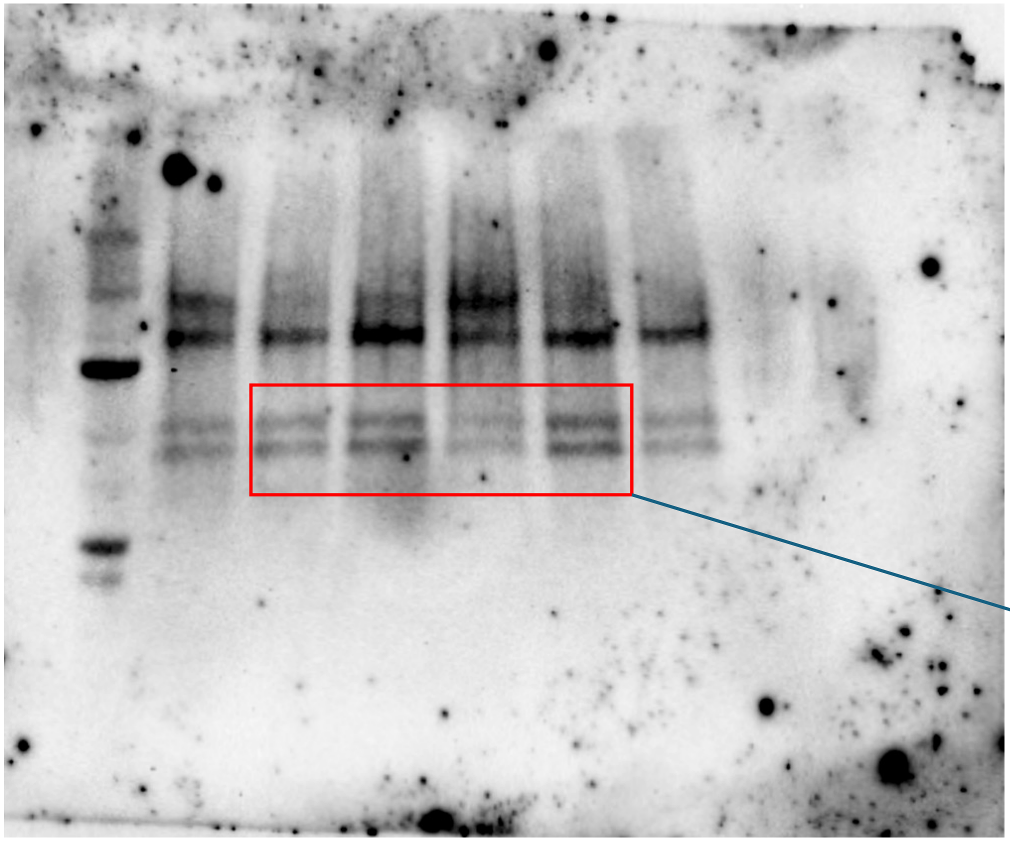

pJNK

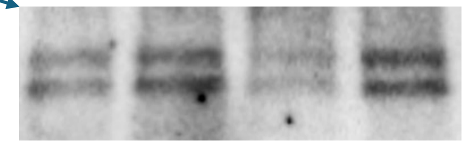

Fig 5e

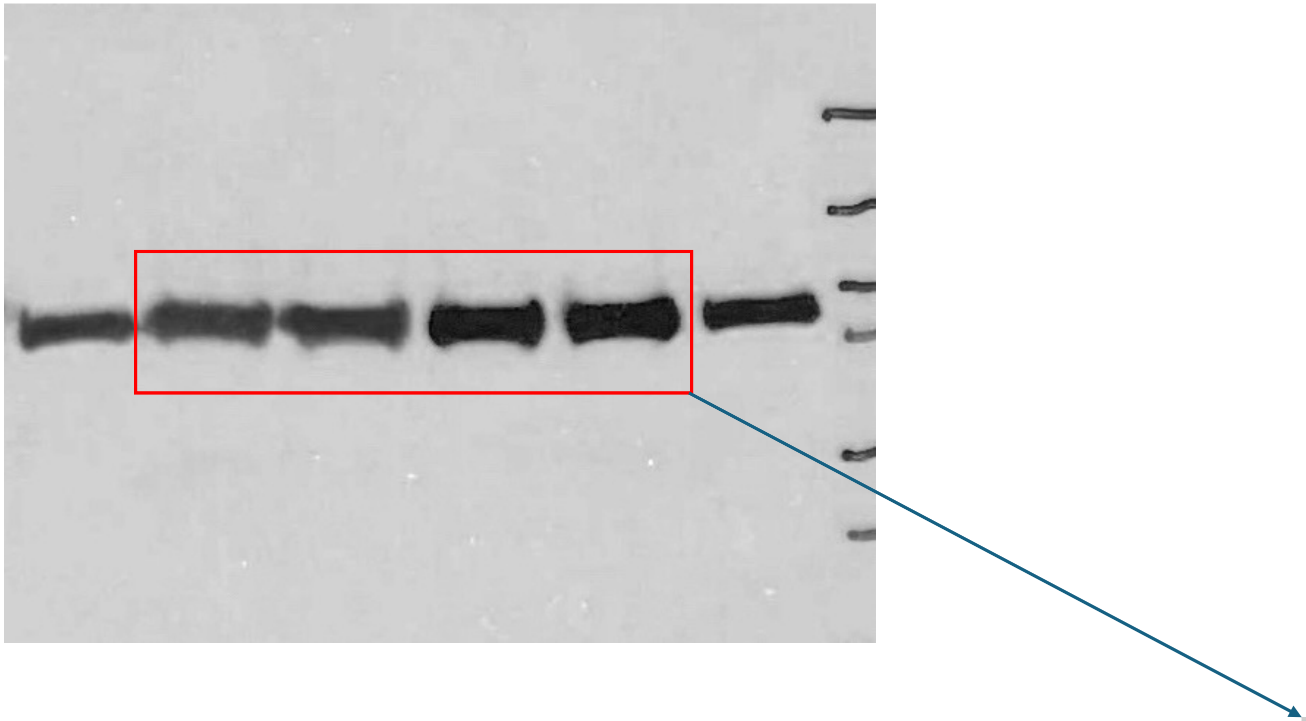

ACTIN

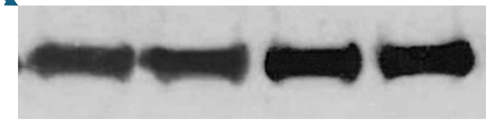

Fig 5j

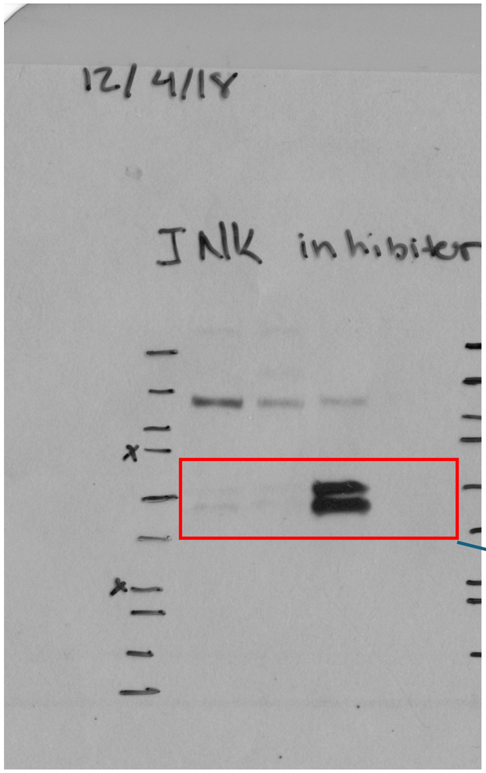

Lane 1-MWM
Lane 2-Grp78$^{f/f}$ Ad-LacZ+DMSO
Lane 3-Grp78$^{f/f}$ Ad-LacZ+JNK Inh
Lane 4-Grp78$^{f/f}$ Ad-iCre+DMSO
Lane 5-Grp78$^{f/f}$ Ad-iCre+JNK Inh
Lane 6-MWM

p-JNK

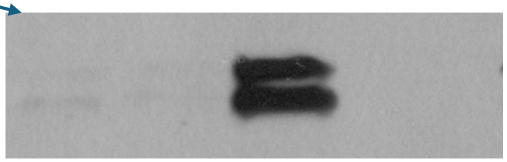

Fig 5j

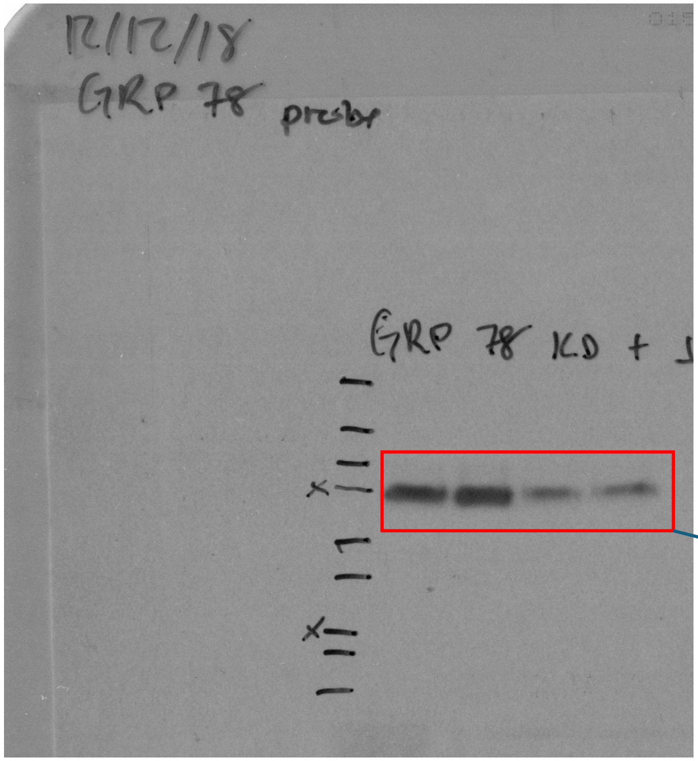

GRP78

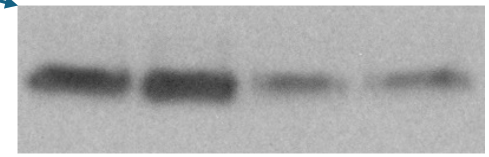

Fig 5j

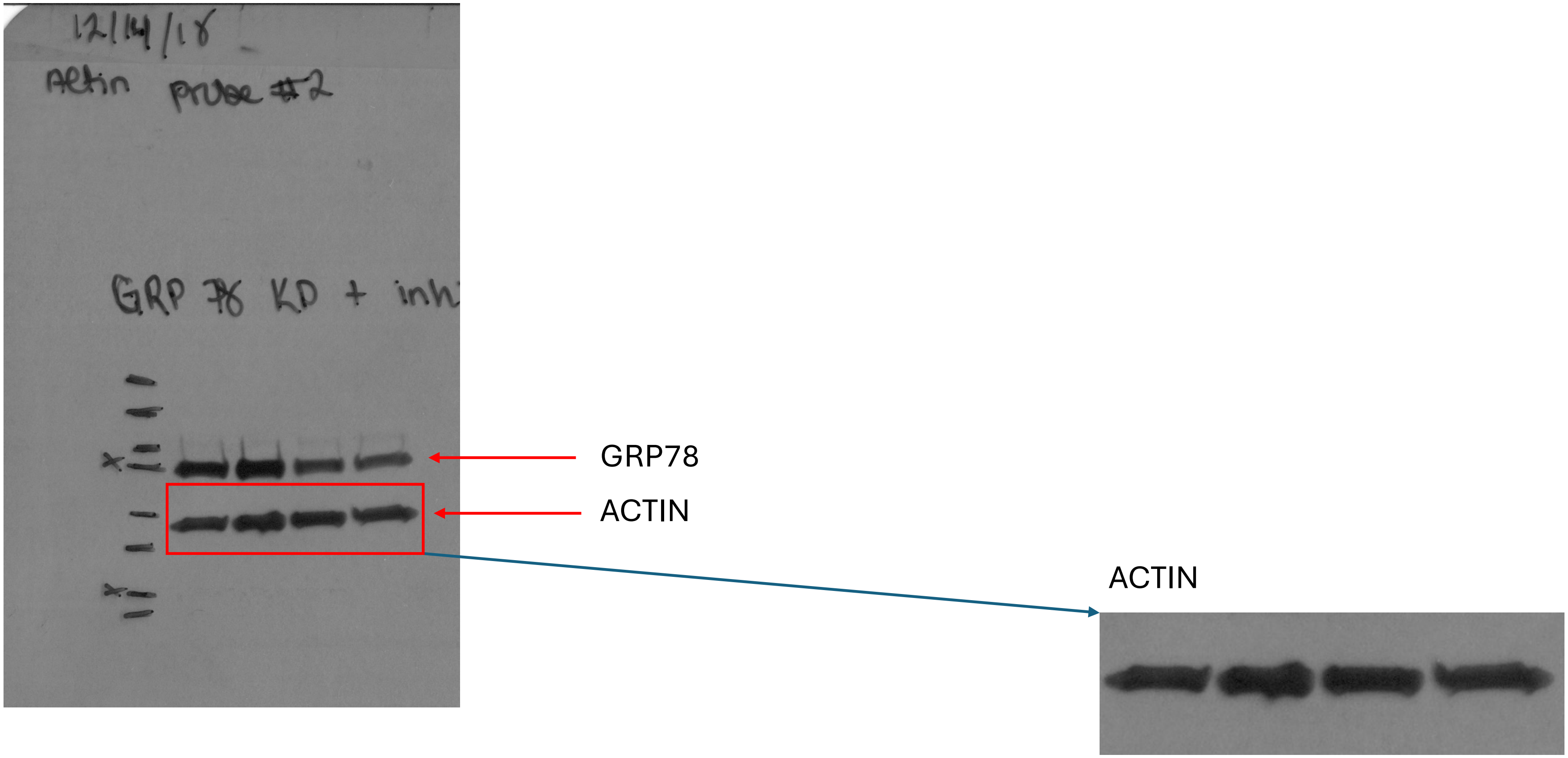

Supplement: Unedited blot and gel images [file jci-136-193035-s024.pdf]
